# Exploring distribution and genomic diversity of begomoviruses associated with yellow mosaic disease of legume crops from India, highlighting the dominance of mungbean yellow mosaic India virus

**DOI:** 10.3389/fmicb.2024.1451986

**Published:** 2024-08-27

**Authors:** Mohammad Akram, Naimuddin Kamaal, Aditya Pratap, Deepender Kumar, Abdul Muin, P. R. Sabale, Revanasidda Aidbhavi, Sunil Kumar Sunani, Meenal Rathore, Sanjeev Gupta, N. P. Singh, N. Dey, G. P. Dixit, Ramakrishnan M. Nair

**Affiliations:** ^1^ICAR-Indian Institute of Pulses Research, Kanpur, India; ^2^ICAR-IIPR Regional Research Station, Dharwad, India; ^3^ICAR-IIPR Regional Research Center, Khurda, India; ^4^ICAR, Krishi Bhavan, New Delhi, India; ^5^Banda University of Agriculture and Technology, Banda, India; ^6^Institute of Life Sciences, Bhubaneshwar, India; ^7^World Vegetable Center, South Asia, Hyderabad, India

**Keywords:** begomoviruses, legumoviruses, diversity, agro-climatic zones, distribution, map

## Abstract

Yellow mosaic disease (YMD) caused by several begomoviruses is one of the major constraints of over a dozen leguminous crops worldwide, particularly in Asian and Southeast Asian countries. The present study aimed to investigate the distribution, diversity and prevalence of begomoviruses associated with YMD in leguminous hosts in five agro-climatic zones of India, to assess the extent of their geographical presence and develop location and crop-specific distribution maps. One hundred and seventy-four leguminous plant samples were tested from 32 locations in India to detect YMD-causing viruses. Additionally, publicly available data were incorporated into this study to provide a comprehensive overview of their distribution in India. This resulted in 581 reports on the DNA-A component representing 119 locations, which were also utilized to depict the distribution of YMD-causing viruses on a map of India. In this study, 117 full-length DNA-A and 103 DNA-B components were successfully characterized, representing the detected mungbean yellow mosaic India virus (MYMIV), mungbean yellow mosaic virus (MYMV), and horsegram yellow mosaic virus in the collected samples. Phylogenetic analysis of isolates of these species showed no differentiation based on location in India. Diversity indices revealed the abundance (55.9%) and dominance (0.56) of MYMIV across 119 locations. These findings hold significant implications for legume researchers, offering insights into disease prevalence and geographic distribution. Furthermore, the distribution of YMD-causing viruses in different agro-climatic zones will help researchers in developing zone-specific YMD-resistant cultivars of the legume crops and would facilitate effective disease management options.

## 1 Introduction

Several leguminous crops, like mungbean (*Vigna radiata*), urdbean (*Vigna mungo*), cowpea (*Vigna unguiculata*), horsegram (*Macrotylopma uniflorum*), mothbean (*Vigna aconitifolia*), soybean (*Glycine max*), common bean or French bean or rajma (*Phaseolus vulgaris*) and pigeonpea (*Cajanus cajan*) are susceptible to yellow mosaic disease (YMD) are caused mostly by bipartite begomoviruses. YMD was initially described in cowpea from Lyllpur (now in Pakistan) (Vasudeva, 1942), and subsequently reported to infect *Phaseolus lunatus* and *Dolichos lablab* in India (Capoor and Varma, 1948). The disease was further recorded in mungbean from New Delhi by Nariani (1960) who termed the putative causal agent as mungbean yellow mosaic virus (MYMV). Subsequently, YMD was also documented in urdbean, mungbean, pigeonpea, horsegram, and French bean (Williams et al., 1968; Nene, 1973; Singh, 1979), and also in wild *Vigna* accessions (Naimuddin et al., 2011a,b,c; Gautam et al., 2014). It has also been reported from other countries such as Sri Lanka, Bangladesh, Philippines, Pakistan, Myanmar, Thailand, Nepal, Indonesia, Malaysia, and Taiwan (Karthikeyan et al., 2014; Mishra et al., 2020). The extent of losses due to YMD depends on the crop stage at the time of infection and the disease intensity. In susceptible varieties, early-stage infections can sometimes lead to complete yield loss (Gill and Singh, 1999). It is estimated that there is an annual loss exceeding US $300 million due to YMD in mungbean, urdbean, and soybean (Varma, 1992). This loss estimate dates back to three decades, which is perhaps even more at present.

Several studies reported that the YMD in leguminous crops is transmitted by whiteflies. An attempt was made to identify the YMD-causal virus by Scanning Electron Microscopy revealed the presence of geminate-like particles (Honda et al., 1983). Furthermore, various studies demonstrated similar geminate particles associated with YMD-affected legumes, confirming that the causal agent is a geminivirus (Muniyappa et al., 1987; Raj et al., 1989; Varma, 1992). Shortly after the full genome sequence of MYMV causing YMD in mungbean from Thailand (Morinaga et al., 1990, 1993), the genome of the virus causing YMD in urdbean was also characterized from north India (Verma et al., 1991). Nevertheless, upon comparing the sequences of these two MYMV isolates responsible for YMD, it became evident that significant variations were surpassing the demarcation limit set for begomovirus species (Fauquet et al., 2008). As a result, the isolate from North India was classified as a separate species and given the name mungbean yellow mosaic India virus (MYMIV).

Later, Barnabas et al. (2010) characterized horsegram yellow mosaic virus (HgYMV) causing YMD in horsegram which was a distinct species differing from both MYMIV and MYMV. Other viruses causing yellow mosaic disease in leguminous plants include dolichos yellow mosaic virus (DoYMV) infecting dolichos (Akram et al., 2015), Rhynchosia yellow mosaic virus (RhYMV) (Ilyas et al., 2009) and Rhynchosia yellow mosaic India virus (RhYMIV) infecting *Rhynchosia minima* (Jyothsna et al., 2011), velvet bean severe mosaic virus (VbSMV) infecting velvet bean (*Mucuna pruriens*) (Zaim et al., 2011), kudzu mosaic virus (KuMV) infecting kudzu (*Pueraria montana*) (Ha et al., 2008), soybean mild mottle virus (SbMMV) and soybean chlorotic blotch virus (SbCBV) infecting soybean (Alabi et al., 2010), Desmodium mottle virus (DeMV) infecting *Desmodium* species (Mollel et al., 2017) and *Cajanus scarabaeoides* yellow mosaic virus (CsYMV) infecting *Cajanus scarabaeoides*, one of the closest wild relatives of cultivated pigeonpea that was found highly similar to RhYMV (Dokka et al., 2023). With the latest addition of CsYMV, a total of 12 distinct begomovirus species have been reported to date to cause yellow mosaic disease in different leguminous hosts. These begomovirus species are phylogenetically distinct from other begomoviruses and collectively named Legume Yellow Mosaic Viruses (LYMVs) or ‘legumoviruses’ (Qazi et al., 2007; Alabi et al., 2010). Henceforth, the term “legumoviruses” will be used to refer the group viruses associated with YMD of leguminous hosts.

The genomes of legumoviruses are bipartite, i.e., consisting of two single-stranded circular DNA molecules named DNA-A (~2.7 kb) and DNA-B (~2.6 kb). DNA-A contains all the information necessary for the replication and encapsidation of the virus (Rogers et al., 1986; Sunter et al., 1987); however, DNA-B is also required for infectivity (Hamilton et al., 1983; Stanley, 1983). Among the legumoviruses, the DNA-B component of SbMMV has not been reported (Alabi et al., 2010). The genes in DNA-A and DNA-B are separated by an intergenic region (IR) that includes a segment of ~200 nucleotides called the common region (CR), a highly conserved region between both molecules. There are six to seven open reading frames (ORFs) in DNA-A and two ORFs in DNA-B of legumoviruses. In DNA-A, two ORFs (AV1 and AV2 coding for coat protein and pre-coat protein, respectively) are in virion-sense orientation and four to five (AC1 to AC4/5) are in the complementary sense orientation. AC1 codes for replication initiator protein (*rep*), AC2 for transcription activator protein (*TrAP*) and AC3 for replication enhancer protein (*REn*). The role of AC4 protein in legume infecting begomoviruses is not clear (Qazi et al., 2007; Ilyas et al., 2009), but in the case of other begomoviruses, it is considered to be involved in symptom determination and countering the host antiviral response to the Rep expression (Hanley-Bowdoin et al., 1999). The AC5 is commonly present in legumoviruses play key roles in the infection process in plants and have RNA silencing suppression activity (Li et al., 2015). In DNA-B, there are two ORFs, the BV1 in virion sense and the BC1 in complementary sense orientation. BV1 codes for the nuclear shuttle protein which is required for the movement of viral DNA (both ss- and dsDNA) between the nucleus and cytoplasm of host cells, whereas BC1 codes for the movement protein which regulates the cell-to-cell movement of the virus through plasmodesmata. It has been demonstrated that BV1 is also involved in long-distance movement of the virus by allowing the spread of the virus through a vascular system of the host (Lazarowitz et al., 1992; Hanley-Bowdoin et al., 1999).

The yellow mosaic disease cycle encompasses a range of cultivated plant species and weeds that serve as hosts, ensuring the availability of inoculum throughout the year (Naimuddin et al., 2014; Bhanu et al., 2015; Marabi et al., 2017, 2021; Chowdary et al., 2022). Differences in disease incidence are influenced not only by the prevalence of alternate hosts but also by the population fluctuation of the polyphagous vector, whitefly due to the weather conditions (Naimuddin et al., 2016). India has a wide range of agro-climatic conditions that change within a span of 500–1,000 km. These agro-climatic zones have distinct weather patterns, which determine the cultivation of specific crops based on the prevailing weather conditions. Many pulse crops are adaptable to different climates and can be grown in various weather conditions. Additionally, certain improved cultivars can thrive in multiple agro-climatic zones simultaneously. As a result, pulse crops are present throughout the year in different zones across the country. In north India, crops like mungbean, urdbean, cowpea, and pigeonpea are cultivated in the spring/summer and *Kharif* seasons. Long-duration pigeonpea, available in fields until April can serve as a source of primary inoculum for spring/summer crops. Winter-season common beans can also act as a source of primary inoculum for spring/summer crops. Once introduced, the virus remains in the crops and it spreads to subsequent *Kharif* crops via the vector, whitefly (Naimuddin et al., 2016).

It is further interesting to note that the *Vigna* crops *viz.*, mungbean, urdbean, cowpea, and ricebean are grown in one or the other part of India throughout the year, ensuring a spatial as well as temporal continuity in the availability of crops as hosts for the pathogen. For example, in northern and part of central India, the spring ecology of these crops includes the crop growth period from February to the last week of April after the harvest of potato, rapeseed-mustard, sugarcane, and pea. The summer crops are sown in the last week of March after the harvest of wheat and chickpea in northern India and harvested in June. During the *Kharif* season, these crops are sown from the first week of July to the first week of November across the country, depending upon the agro-climatic region. In south Indian states, the *rabi* crops are usually sown in the months of October–November and January as sole and/or inter-crops. Besides this, there are special ecologies such as relay cropping in rice fallows in peninsular India, where crops are sown in the standing matured paddy crop just before harvest.

However, it is also evident that there are great differences in the climatic conditions, rainfall patterns, and soil types across different regions of the country. Based on these variations, the country has been divided into different agro-climatic zones wherein each zone comprises multiple locations to represent micro-climates within the zones. The new varieties of pulse/legume crops, along with their production and protection technologies, are evaluated at various locations through the All India Coordinated Research Project (AICRP) on pulses. Within the AICRP framework, it is mandatory to exchange the advanced breeding materials/genotypes across different locations. The challenge arises while assessing the same materials against all species/strains of an unidentified YMD-causing virus(es) at any given location. This presents a significant barrier to the development of new YMD-resistant cultivars. Therefore, to ensure that only highly YMD-resistant varieties are released for cultivation across diverse locations, it is critical to gather comprehensive information on the prevalence of different virus species associated with YMD across various agro-climatic zones.

It has been observed that the improved varieties of cultivated crops like mungbean, urdbean, cowpea, soybean, horsegram, and mothbean show temporal and spatial variation in the YMD development (Naimuddin et al., 2016; Mishra et al., 2020; Project Coordinator Report, 2022). This could probably be attributed to the overlapping cropping of legume crops, variations in weather conditions, fluctuations in the vector population, and the prevalence of different virus species at different locations. As of now, it is generally considered that MYMV and HgYMV are present in the southern part of India and MYMIV is present in the north and central parts of the country (Varma and Malathi, 2003; Usharani et al., 2004). This study aimed to investigate the diversity, prevalence, and geographical distribution of viruses associated with YMD in leguminous/weed hosts within the country, resulting in the development of location and crop-specific distribution maps to facilitate a comprehensive understanding of their spread.

## 2 Materials and methods

### 2.1 Survey and sample collection

Field surveys were conducted during 2018–2023 at 38 distinct locations representing five agro-climatic zones for legume crops in India. The zoning system has been adopted based on agro-climatic parameters such as annual rainfall, temperature, relative humidity, and water resources (Table 1). The zoning, of course, does not exclude the possibility of overlapping crops in different seasons, for example, spring and summer, thereby providing an ample opportunity for the YMD-causing pathogen to thrive well and perpetuate across crops, seasons, and agro-climatic zones. A total of 259 YMD-affected samples representing different legume crops were collected. The collections were made at random and solely based on the disease symptoms observed irrespective of the cultivars. From each location, 1–8 fields were surveyed for the affected plant leaves and the average percent disease incidence (%) was noted. During survey, a 1-meter row is randomly selected at 5 different locations within the selected field. The total numbers of plants in each selected row, including both healthy and diseased plants, were counted. Percent Disease Incidence (PDI) is then calculated using the following formula:


$$PDI=\left(\begin{array}{l} \mathrm{Number}\;\mathrm{of}\;\mathrm{affected}\;\mathrm{plants}\;\mathrm{in}\;5\;\mathrm{rows}/\\ \mathrm{Total}\;\mathrm{number}\;\mathrm{of}\;\mathrm{plants}\;\mathrm{in}\;5\;\mathrm{rows} \end{array}\right)\times 100$$

Most of the samples were collected in person, while some of them were obtained from volunteers via Indian postal services. In both cases, leaf samples were surface cleansed before bagging them in perforated paper envelopes to avoid sample spoilage during transit. The disease-affected leaf samples were brought to the laboratory and processed at the ICAR-Indian Institute of Pulses Research (ICAR-IIPR), Kanpur, Uttar Pradesh, India.

**Table 1:** Details of weather conditions, percent sample collection and viruses detected in five agro-climatic zones of India.

Agro-climatic zones	States by zone	Locations (number of samples)*	Average temp. (min-max)	Annual rainfall range	Relative humidity range (in %)	Major pulse/legume crops grown	Crops samples tested (count)	Zonewise samples tested (%)^a^	Virus detected (in %)^a^
Central Zone (CZ)	Maharashtra, Madhya Pradesh, Chhattisgarh, Gujarat	Bhopal (9), Devas (4), Harda (5), Hoshangabad (5), Indore (5), Jalna (2), Parbhani (2), Phanda (4), Sehore (5), Obaidullaganj (2)	In summer: 25 to 40°C In winter: 10 to 25°C	25–100 cm	In summer: 30–60 In winter: 20–50	Soybean, chickpea, pigeonpea, mungbean, urdbean, cowpea, fieldpea, lathyrus	Mungbean (39), urdbean (4)	24.71	MYMIV = 22.41; MYMV = 1.72; MYMIV+MYMV = 0.57
North West Plain Zone (NWPZ)	Western Uttar Pradesh, Northern Rajasthan, plains of Uttarakhand, Punjab, Haryana, New Delhi and the Jammu region of Jammu and Kashmir	Hisar (16), Jobner (1), Jodhpur (3), Ludhiana (12), New Delhi (7)	In summer: 26 to 44°C In winter: 5 to 23°C	75–150 cm	In summer: 20–50 In winter: 15–40	Chickpea, lentil, pigeonpea, mungbean, urdbean, mothbean, cowpea, fieldpea	Mothbean (4), mungbean (24),urdbean (9), cowpea (2)	22.41	MYMIV = 17.24; MYMIV+MYMV = 4.59; MYMIV+MYMV+HgYMV = 0.57
North East Plain Zone (NEPZ)	Central and Eastern Uttar Pradesh, Bihar, Jharkhand, Assam, West Bengal	Banda (1), Faizabad (2), Kanpur (16), Varanasi (2)	In summer: 26 to 41°C In winter: 4 to 24°C	100–200 cm	In summer: 50–90 In winter: 40–70	Mungbean, chickpea, lentil, pigeonpea, urdbean, lathyrus, fieldpea	Mungbean (10), urdbean (6), cowpea (6), horsegram (1)	12.06	MYMIV = 11.49; MYMIV+MYMV = 0.57
South Zone (SZ)	Tamil Nadu, Andhra Pradesh, Karnataka, Kerala, Telangana, Odisha, Andaman & Nicobar islands	Bagalkot (3), Belgaum (8), Bengaluru (3), Bhubaneshwar (5), Coimbatore (6), Dharwad (16), Hyderabad (3), Khorda (3), Namakkal (6), Raichur (5), Vamban (10)	In summer: 25 to 35°C In winter: 15 to 25°C	50–300 cm	In summer: 50–80 In winter: 40–70	Pigeonpea, urdbean, mungbean, chickpea, cowpea, horsegram	Cowpea (16), mungbean (25), mothbean (5), urdbean (12), horsegram (4), *V. stipulacea* (1)	39.65	MYMIV = 7.47; MYMV = 10.34; HgYMV = 3.44; MYMIV+MYMV+HgYMV = 2.87; MYMIV+MYMV = 8.05; MYMIV+HgYMV = 0.57; MYMV+HgYMV = 6.89
North Hill Zone (NHZ)	Tripura, Arunachal Pradesh Manipur, Nagaland, Meghalaya, Sikkim, Jammu & Kashmir, Upper part of Uttarakhand and Himachal Pradesh	Pantnagar (1), Srinagar (1)	In summer: 5 to 30 ° C In winter: −4 to 24°C	150–200 cm	In summer: 40–70 In winter: 30–60	French bean, lentil, chickpea, mungbean, urdbean, fieldpea	Mungbean (1), cowpea (1)	1.14	MYMIV = 1.14

*Locations from where samples (Total = 174) tested by PCR assay in this study.

a Total sample size = 174.

### 2.2 DNA isolation and PCR-based detection of legumoviruses

Dried (20–50 mg) and/or fresh leaf samples (100 mg) were crushed with the aid of liquid nitrogen and processed for DNA extraction using DNeasy Plant Mini Kit (QIAGEN, GmbH, Hilton, Germany) following the manufacturer’s protocol. The quality of extracted DNA was checked on 1% agarose gel electrophoresis and was used as a template in the PCR-based detection of four begomoviruses (MYMIV, MYMV, DoYMV, and HgYMV). PCR assays were conducted in a 25 μL reaction mixture that consisted of 1 μL template DNA, 12.5 μL of DreamTaq PCR Master Mix (2X) (Thermo Scientific, Germany), 1 μL (25 pmol) each of forward and reverse primer, and 9.5 μL nuclease-free water. The thermal profile for the PCR involved initial denaturation at 95°C for 3 min followed by 40 cycles of denaturation at 95°C for 30 s, annealing at 56°C for 30 s, and elongation at 72°C for 60 s, with a final elongation step at 72°C for 5 min. The primers used in the present study were designed (Kumar et al., 2024) to amplify a portion of DNA-A covering the coat protein (CP) gene (AV1) (Supplementary Table S1). The PCR products were observed on 1% agarose gel electrophoresis and results were validated by commercially sequencing primer-specific amplicons followed by BLAST (blastn) analysis with default parameters.^1^ Sequences generated in the present study were submitted to GenBank.^2^

### 2.3 Rolling circle amplification and full genome sequence analyses

The samples found positive in PCR-based detection for any of the four YMD-causing viruses were subjected to rolling circle amplification (RCA) using REPLI-g Mini Kit (QIAGEN, GmbH, Germany), and the product obtained was diluted following the manufacturer’s protocol. The diluted RCA products were used as templates for PCR-based amplification of full-length DNA-A and DNA-B components. Fourteen primer pairs targeting DNA components of MYMIV (DNA-A and DNA-B), MYMV {DNA-A, DNA-B(1) and DNA-B(2)} and HgYMV (DNA-A and DNA-B) and corresponding PCR conditions were used as described in the previous studies (Akram et al., 2020, 2022). The amplicons were observed on 1% agarose gel electrophoresis and then sequenced through a sequence service provider (Genematrix LLP, Pune, India). Sequence data were assembled and analyzed with the aid of BioEdit v7.2 (Hall, 1999). The assembled full-length sequences of DNA-A and DNA-B components of the identified begomoviruses were characterized with the aid of ORF Finder^3^ followed by BLAST (blastp) and then submitted to GenBank.

### 2.4 Data retrieval from public database and compilation

Accessions with complete and partial sequences of the DNA-A and DNA-B components of the 12 legumovirus isolates (CsYMV, DeMV, DoYMV, HgYMV, KuMV, MYMIV, MYMV, RhYMV, RhYMIV, SbCBV, SbMMV, and VbSMV) available in NCBI Nucleotide database^4^ till February 2024 were accessed, retrieved and used for extracting information about their host and location for consideration in the present study after confirmation of each accession by BLAST (blastn) analysis with default parameters. The analysis involved 528 and 267, publicly available accessions of DNA-A and DNA-B components, respectively (Supplementary Table S3). This revealed discrepancies in the information on various accessions of MYMV and MYMIV available in the public database. The accession AJ867554, submitted as DNA-B of MYMV, is DNA-B of HgYMV. Further, the accession MH885653 is reported as the coat protein gene of MYMIV, but the BLAST results indicate its affiliation with MYMV. The accessions AJ315469 and AJ315667 are available in the database as partial sequences of the soybean yellow mosaic virus, which are affiliated with MYMIV. The accession AJ315963 wrongly designated as the soybean yellow mosaic virus CP gene is the CP gene of MYMV. The accession AJ315668 is inaccurately named as the AC5 gene of the soybean yellow mosaic virus, but this is also affiliated with MYMIV. AJ582267 is inaccurately named as soybean yellow mosaic virus DNA-B; it is, in fact, MYMV DNA-B. AJ315666, erroneously named as soybean yellow mosaic virus movement protein (MP) gene (BV1), is the MP gene of MYMIV. All these inaccuracies in the NCBI database were rectified before being used in the present study.

The sequence information generated in this study on full and partial sequences of DNA-A and DNA-B components of MYMIV, MYMV, and HgYMV, after assembling and confirming the respective virus species based on results of ORF Finder and BLAST hits, were added to the retrieved information. Thus, the compiled information resulted in a total of 717 and 370 data points (DPs) for DNA-A and DNA-B components, respectively. The frequency distribution of four legumoviruses (MYMIV, MYMV, DoYMV, and HgYMV) reported more often from India across the five agro-climatic zones, was illustrated using Circos (Krzywinski et al., 2009).

### 2.5 Construction of a distribution map of legumoviruses

The information assembled corresponding to legumoviruses’ DNA-A components was used to generate a map depicting the location of each accession. The latitude and longitude coordinates corresponding to each location were collected manually using online tools such as Google Maps^5^ and Latitude and Longitude Finder.^6^ These coordinates were plotted on the map of India using the online tool Google Looker Studio.

### 2.6 Phylogenetic analysis

Full-length (DNA-A and DNA-B) sequences of RefSeq accessions of all the bipartite begomoviruses cataloged at the ICTV (Lefkowitz et al., 2018) taxonomy browser along with the accessions of all the isolates of legumoviruses considered for this study, present at the NCBI GenBank were considered for the phylogenetic analysis. Cowpea golden mosaic virus (CpGMV) often considered as the link between Old World (OW) and New World (NW) begomoviruses was also considered (Alabi et al., 2010; Akram et al., 2022). Maize streak virus (MSV) was considered as an outgroup for the analysis of both DNA-A and DNA-B components. Multiple sequence alignment was performed using the *FFT-NS-2* (default) strategy in MAFFT v7.526 (Katoh and Standley, 2013). MEGA v11 (Tamura et al., 2021) was used to generate a phylogenetic tree with 100 bootstraps using the Maximum Likelihood method and the Tamura-Nei model (Tamura and Nei, 1993) with uniform rates and using all sites. The Tamura-Nei model was selected due to its effectiveness in previous studies involving begomoviruses (Mauricio-Castillo et al., 2014; Torres-Herrera et al., 2019; Gupta et al., 2022; Santosa and Somowiyarjo, 2023) and its ability to account for heterogeneous nucleotide substitution rates, including both transitions and transversions, making it suitable for our dataset. The phylogenetic trees were visualized using iTOL v6 (Letunic and Bork, 2024).

### 2.7 Estimation of diversity indices

Diversity indices were computed based on DNA-A-related data of viruses to ascertain their species diversity, abundance, dominance, richness, and evenness, within or between community (host plants) and habitats (geographical location). The information on reports from India was further categorized into the number of the host plant species and the locations they occurred at, as well as the total number of reports as DPs, within a particular defined community and habitat. Apart from this, the indices were also calculated on major host plant species that had more than one virus species detected/reported.

The relative abundance of virus species was estimated by counting the proportion of reports of each species to the total reports of all the species combined. It was expressed as the percent abundance of each species relative to the total species in a particular defined community or habitat. The species diversity was calculated using the Shannon-Weaver Index:


$$H^{\prime }=-\overset{S}{\underset{i=1}{\sum }}p_{i}\times \ln p_{i}$$

where “*p_i_*” is the proportion of reports of the *i*^th^ (*i* = 1, 2,…, *S*) species of virus among the total reports of all the virus species combined. The *H′* of each virus species was interpreted in relation to the total species in a particular defined community or habitat (Shannon and Weaver, 1963). The Effective Number of Species (ENS) is a measure of true diversity that aids in comprehending the actual number of species in a community or habitat, particularly when all species are equally abundant (Jost, 2006). True diversity is described as “the number of equally common species needed to produce a specific index value.” ENS was determined by the formula (MacArthur, 1965),


$$D=\exp\left(H\right)$$

The dominance of virus species within a given community or habitat was ascertained using the Berger-Parker dominance index “d” (Berger and Parker, 1970). This index provides insight into the proportion of a particular virus species in relation to the total number of species present in the environment. It was calculated with the formula:


$$d=n_{i}/NT$$

where *n_i_* represents the number of reports for the *i*^th^ species and NT represents the total number of reports within the community or habitat. The reciprocal of the dominance index, i.e., 1/d , implies that an increase in the “d” value indicates an increase in the diversity of species in a particular community/habitat and a decrease in the dominance of a particular species.

To assess species richness, Margalef’s index (*D_mg_*), the most fundamental measure of biodiversity, was utilized (Margalef, 1973). This index represents the count of the occurrence of particular species distributed in a specific community or habitat. It was calculated using the formula:


$$D_{\mathrm{mg}=\left(S-1\right)/\ln N}$$

where ‘*S*’ is the number of species recorded from a community or habitat of interest and “N” is the total number of records combined of all “*S*” species. The species evenness index, which measures the relative abundance of each species in a community/habitat, was used to determine the closeness of density of each virus species to others in a defined habitat or community. If each species is equally abundant, evenness is one. This index was computed using the following formula (Pielou, 1966):


$$E=H^{\prime }/\ln S$$

where *H′* represents the Shannon diversity index and *S* is the total number of species in the habitat or community.

## 3 Results

### 3.1 Sample collection and PCR assays

**Figure 1:**
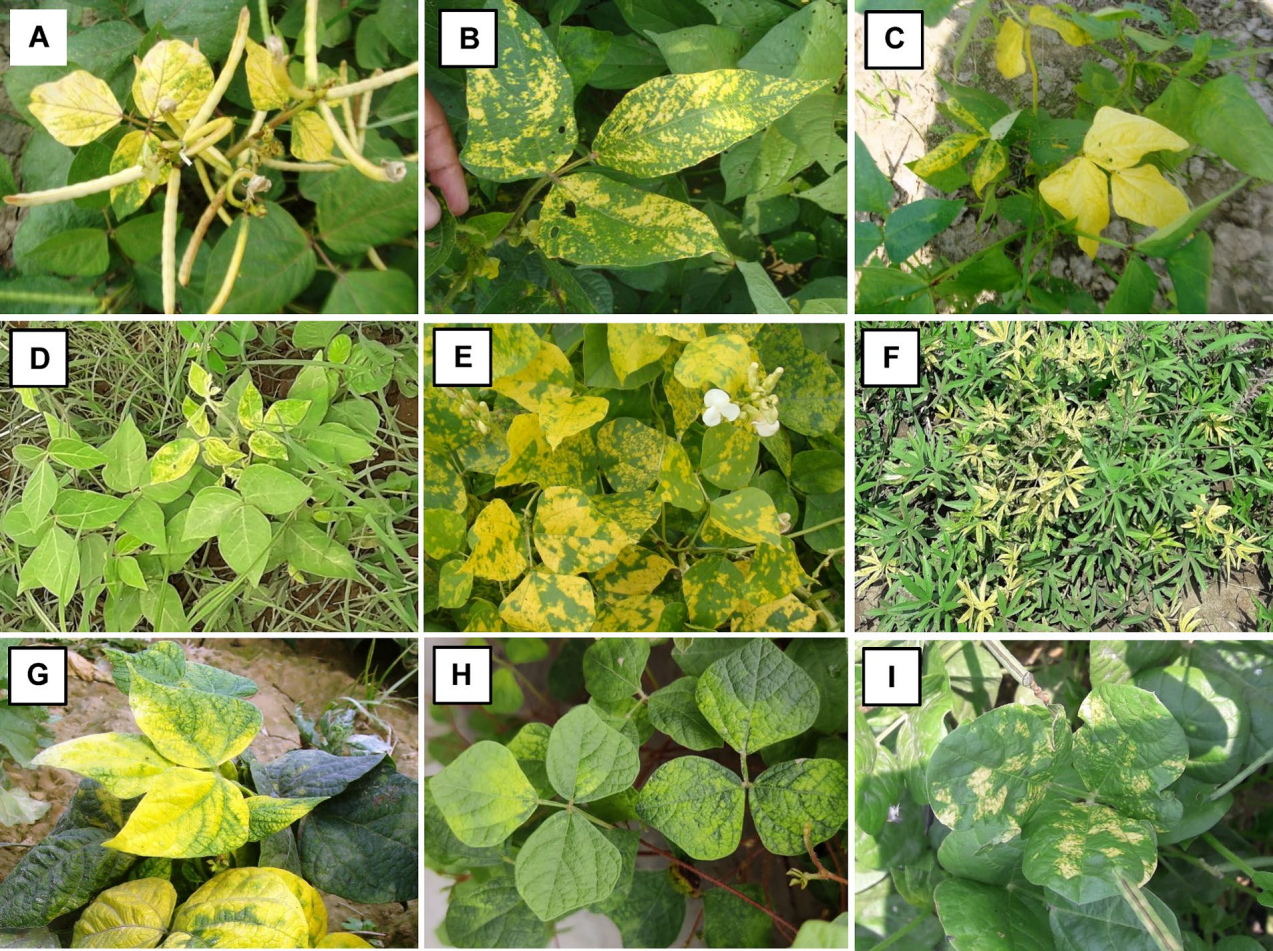
Representative illustration showing symptoms of yellow mosaic disease observed in **(A)** mungbean; **(B)** urdbean; **(C)** cowpea; **(D)** horsegram; **(E)** dolichos; **(F)** mothbean; **(G)** French bean; **(H)** *Rhynchosia minima* and **(I)** *Vigna stipulacea,* collected from different locations in India.

A total of 259 symptomatic leaf samples representing six pulse crops including mungbean (*n* = 148), urdbean (*n* = 69), cowpea (*n* = 26), horsegram (*n* = 5), and mothbean (*n* = 10) along with the wild relative *Vigna stipulacea* (*n* = 1) were processed from 38 different locations in India for this study. The overall disease incidence in all the fields surveyed fell within the range of 5 to 60% (Figure 1; Supplementary Table S2). The collected leaf samples were processed for DNA extraction, where 236 samples yielded DNA when observed on agarose gel electrophoresis. Given a total of 236 samples now representing 32 locations, with multiple samples taken from each location, only 174 out of the 236 samples with good quality isolated DNA were subjected to PCR-based detection of the four legumoviruses *viz.* MYMIV, MYMV, DoYMV, and HgYMV to keep this study cost-effective. This was done by compromising none of the 32 locations (Supplementary Table S2). The maximum number of samples were from SZ representing 39.65% of the samples, followed by 24.71% from the CZ, 22.41% from the NWPZ, 12.06% from the NEPZ and lastly, 1.14% from the NHZ (Table 1).

Prior to subjecting the 174 samples to the PCR assays, the designed primers specific to MYMIV, MYMV, DoYMV and HgYMV were tested on 71 YMD-affected samples comprising different hosts namely mungbean (*n* = 9), urdbean (*n* = 6), cowpea (*n* = 26), rajma or French bean (*n* = 21) and weeds (*n* = 9), collected from the premises of ICAR-IIPR, Kanpur. Sequences of primer-specific amplicons subjected to BLAST analysis revealed high similarity with the corresponding targeted virus species. The sequences of these amplicons were trimmed to obtain the AV1 gene of 774 bp, present on the DNA-A component, and submitted in GenBank (Supplementary Table S3).

Of the selected 174 samples, 104 samples tested positive for MYMIV comprising mungbean (*n* = 61), urdbean (*n* = 27), cowpea (*n* = 13), mothbean (*n* = 2), and horsegram (*n* = 1). Similarly, 21 samples comprising mungbean (*n* = 17), mothbean (*n* = 2), and cowpea (*n* = 2) tested positive for MYMV. Five samples comprising horsegram (*n* = 3) and mothbean (*n* = 2) tested positive for HgYMV. The remaining 44 samples showed mixed infection, where 24 samples including mungbean (*n* = 16), urdbean (*n* = 5), cowpea (*n* = 2), and mothbean (*n* = 1) were found positive with MYMIV + MYMV. Likewise, 12 samples comprising mungbean (*n* = 7), mothbean (*n* = 2), cowpea (*n* = 2), and horsegram (*n* = 1) were found positive with MYMV + HgYMV, and one sample of urdbean with MYMIV + HgYMV. The remaining 7 samples (2 each of cowpea and mungbean; one each of mothbean, urdbean, and *V. stipulacea*) tested positive for mixed infection of MYMIV + MYMV + HgYMV (Supplementary Table S2).

### 3.2 Characterization of full-length DNA-A and DNA-B components

The positively tested 174 samples were further processed for viral titer enrichment using rolling circle amplification (RCA). Collectively, of these 174 samples, 117 full-length DNA-A (~2.7 kb) and 103 full-length DNA-B (~2.6 kb) components of MYMIV, MYMV, and HgYMV from samples representing 28 locations from India were successfully characterized. Good quality sequence reads were not obtained from the samples representing the remaining four locations. However, since these samples tested positive using species-specific primers, these locations were also included in the study. As a result, in this study legumoviruses were detected from a total of 32 locations across India. For the first time, of these 28 locations, based on full-length sequences, we identified HgYMV infecting cowpea from Belgaum, horsegram and mungbean from Dharwad, and urdbean from Raichur. Similarly, MYMIV infecting cowpea from Bengaluru, Ludhiana and Raichur, mothbean from Jodhpur, mungbean from Banda, Bhopal, Coimbatore, Dewas, Dholi, Faizabad, Harda, Hisar, Hoshangabad, Indore, Jodhpur, Namakkal, Obaidullaganj, Phanda, Sehore, Vamban and Varanasi, urdbean from Aligarh, Faizabad, Hisar, Phanda, Raichur, Vamban and Varanasi. MYMV infecting French bean from Bengaluru, mungbean from Bagalkot, Hyderabad and Namakkal, urdbean from Hisar and Raichur. Notably, during this study, first ever reports on simultaneous infection of all three species (HgYMV, MYMV, and MYMIV) infecting mothbean at Belgaum and *Vigna stipulacea* at Raichur were made (Akram et al., 2020, 2022). Virus species-wise number of DNA-A and DNA-B components characterized were: 8 full-length DNA-A component of HgYMV (2,735 bp), 81 of MYMIV (2,741–2,747 bp), and 28 of MYMV (2,725–2,730 bp); 14 full-length DNA-B component of HgYMV (2,668–2,672 bp), 68 of MYMIV (2,653–2,683 bp) and 21 of MYMV (2,656–2,683 bp). These sequences were further submitted to GenBank. The accession numbers of these full-length sequences are provided in Supplementary Table S3.

### 3.3 Data retrieval, compilation, and interpretation

The collected data includes reports on 285 partial and 244 full-length sequences of the DNA-A component, as well as reports on 67 partial and 200 full-length sequences of the DNA-B component of the 12 legumoviruses. This information has been compiled, along with the data generated in this study comprising 71 partial and 117 full-length sequences of DNA-A component, and 103 full-length sequences of DNA-B component, is provided in Supplementary Table S3. A focus on hosts and locations is evident in the compiled information which included 717 reports (or DPs) on the DNA-A component and 370 reports (or DPs) on the DNA-B component. Of these reports (or DPs), 581 are for DNA-A and 287 for DNA-B from India. A country-wise summary statistics based on the compiled information for the DNA-A component of 12 legumoviruses, including the number of reports, hosts, and locations, is provided in Table 2. Except for CsYMV, the binomial nomenclature of these legumoviruses has been approved by the International Committee on Taxonomy of Viruses (ICTV: https://ictv.global/msl) (Table 2). However, in this study, we have used the old nomenclature for these legumoviruses.

**Table 2:** DNA-A component-based summary statistics of the compiled information on 12 legumoviruses identified within India (in bold) as compared to other countries.

Serial no.	Virus	Virus binomial name*	Country	No. of reports	No. of locations	No. of hosts
1.	*Cajanus scarabaeoides yellow mosaic virus*	Not assigned	**India**	1	1	1
2.	Desmodium mottle virus	*Begomovirus desmodii*	Uganda	2	1	1
3.	dolichos yellow mosaic virus	*Begomovirus dolichoris*	Bangladesh	5	4	1
			**India**	43	12	8
4.	horsegram yellow mosaic virus	*Begomovirus loniceramusivi*	**India**	60	30	21
			Sri Lanka	1	1	1
5.	kudzu mosaic virus	*Begomovirus puerariae*	China	6	3	1
			Vietnam	2	2	2
6.	mungbean yellow mosaic India virus	*Begomovirus vignaradiataindiaense*	Bangladesh	4	3	3
			**India**	324	75	24
			Indonesia	26	15	3
			Nepal	5	2	3
			Oman	11	1	4
			Pakistan	41	10	4
7.	mungbean yellow mosaic virus	*Begomovirus vignaradiatae*	Cambodia	1	1	1
			**India**	146	40	26
			Pakistan	8	4	3
			Thailand	2	1	2
			Vietnam	5	1	1
8.	Rhynchosia yellow mosaic India virus	*Begomovirus rhynchosiaindiaense*	**India**	2	1	1
9.	Rhynchosia yellow mosaic virus	*Begomovirus rhynchosiaflavi*	**India**	4	4	4
			Pakistan	2	1	1
10.	soybean chlorotic blotch virus	*Begomovirus glycinepallidi*	Benin	1	1	1
			Cameroon	3	1	3
			Nigeria	6	2	4
			Togo	3	1	3
11.	soybean mild mottle virus	*Begomovirus glycinevariati*	Nigeria	2	2	2
12.	velvet bean severe mosaic virus	*Begomovirus mucunae*	**India**	1	1	1

^*^Renamed by the International Committee on Taxonomy of Viruses (ICTV: https://ictv.global/msl).

### 3.4 Distribution map and diversity of legumoviruses in India

**Figure 2:**
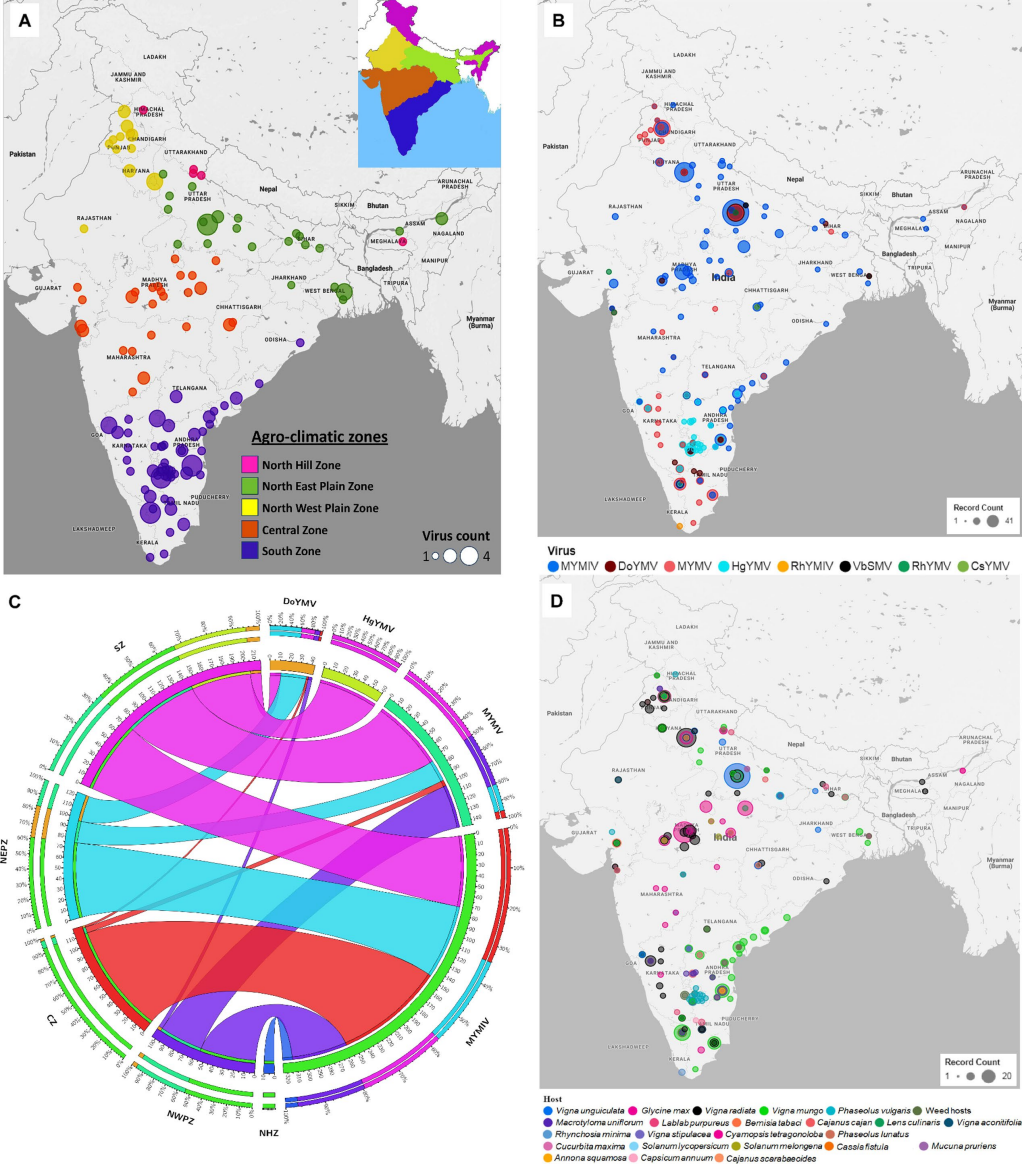
**(A)** An outline map of India showing 119 locations across five agro-climatic zones: North Hill Zone (NHZ), North East Plain Zone (NEPZ), North West Plain Zone (NWPZ), Central Zone (CZ), and South Zone (SZ), where reports of legumovirus were made. An illustration of these five zones in India is shown as an inset at the top right corner. Each location is represented with a circular spot, colour-coded to correspond with the respective zone. The variable size of the spot denotes the detection of one to a maximum of four out of eight virus species from that location; **(B)** The 119 locations from where 8 legume-infecting begomoviruses were detected and reported. Different colours of spots were used to represent the 8 virus species. The variable size of the spot is proportional to the number of reports made per location. Several locations have reports of multiple virus detections and in such cases, the species with the least reports is shown at the centre of the spots shown as concentric circles at such locations. **(C)** A circos plot showing the frequency distribution of reports made on MYMIV, MYMV, DoYMV and HgYMV from CZ, NHZ, NEPZ, NWPZ and SZ. The zones are shown anticlockwise in descending order with respect to the total data points (DPs) from the respective zone. The width of the colored ribbon is directly proportional to the frequency of respective virus species in relation to the corresponding zones. **(D)** Distribution of reports on 8 legumoviruses detected in India across various hosts. There are 22 crop hosts categorized as leguminous and non-leguminous, represented by differently colored spots placed at their respective locations of detection. Additionally, 31 species of weed hosts, are considered as one category and are depicted with a single colored spot. The variable size of each spot indicates the number of reports associated with a particular host, with hosts having the least number of reports at each location depicted at the centre of concentric circles. RhYMV, Rhynchosia yellow mosaic virus; CsYMV, *Cajanus scarabaeoides* yellow mosaic virus; MYMV, mungbean yellow mosaic virus; HgYMV, horsegram yellow mosaic virus; VbSMV, velvet bean severe mosaic virus; RhYMIV, Rhynchosia yellow mosaic India virus; MYMIV, mungbean yellow mosaic India virus; DoYMV, dolichos yellow mosaic virus.

Eight (CsYMV, DoYMV, HgYMV, MYMIV, MYMV, RhYMV, RhYMIV, and VbSMV) out of the twelve identified legumoviruses worldwide have been reported from 119 locations of India distributed across five zones (Figure 2A and Supplementary Figure S1). From several locations in India, more than one species of legumoviruses were detected (Figure 2A; Supplementary Table S4). An illustration of the same is shown in Figure 2B. The virus species namely KuMV, DeMV, SbMMV, and SbCBV have not been reported so far from India.

Among the 8 legumoviruses, CsYMV, RhYMIV, and VbSMV are reported only from single locations *namely* Raipur (CZ), Thiruvananthapuram (SZ), and Lucknow (NEPZ); infecting *Cajanus scarabaeoides*, *R. minima,* and *Mucuna pruriens* (velvet bean), respectively. RhYMV has been reported from 4 different locations in the CZ and NEPZ. HgYMV was observed to have a distribution restricted within the SZ with 60 reports from 30 locations. DoYMV and MYMV were observed in all the zones except for the NHZ with 43 and 146 reports from 12 and 40 locations, respectively. MYMIV was observed throughout the five zones and has 324 reports from 75 locations (Figure 2C,D). The survey conducted in this study alone provides evidence for the predominant existence of three species *viz.* MYMIV, MYMV and HgYMV in the SZ. Furthermore, mixed infections involving any combination of these three species, as well as simultaneous infections of all three, were observed (Table 1).

The type of host described in 581 DPs for 8 legumoviruses from 119 locations can be categorized into a total of 54 hosts consisting of 22 species of crops (legume and non-legume), 31 species of weeds, and whiteflies (Figure 2D). These “weeds” are either ornamental flowering plants commonly used for gardening or plants that are often found naturally occurring near legume crop fields. Among the legume crops, MYMIV and MYMV were most frequently detected in the mungbean, followed by urdbean and soybean. To some extent, MYMIV and MYMV were also reported in cowpea and French bean (Supplementary Figure S2). Other legume crops such as lentil, clusterbean, pigeonpea, mothbean, dolichos, and horsegram were reported as hosts for either MYMIV or MYMV or both. Maximum reports of DoYMV have been reported from dolichos, followed by cowpea and rajma, with only two reports from mungbean. Urdbean, however, has not been reported to be infected by the DoYMV so far. Maximum reports of HgYMV have been from French bean followed by horsegram and pigeonpea. It was also reported on other legumes such as mungbean, urdbean, soybean, limabean, cowpea and mothbean (Supplementary Figure S2). Non-legume crops such as *Cucurbita maxima*, *Solanum lycopersicum*, *Annona squamosal* and *Solanum melongena* have also been reported to carry the infection of MYMIV, whereas DoYMV is reported infecting another non-legume crop *Capsicum annum*. In the case of whiteflies, reports were only made for the detection of MYMIV. The absence of reports of other legumoviruses in whiteflies may appear misleading, as it is well documented that all legumoviruses are transmitted by whiteflies and therefore this information appears to be insufficient for understanding the distribution of virus species. There have been 45 reports in total, documenting infections in 31 distinct weed hosts in India. Among these reports, MYMIV accounts for 9, MYMV for 19, DoYMV for 5, HgYMV for 11, and RhYMIV for 1 representing 17 locations (Supplementary Tables S3, S4).

### 3.5 Phylogenetic relationship amongst bipartite begomoviruses

**Figure 3:**
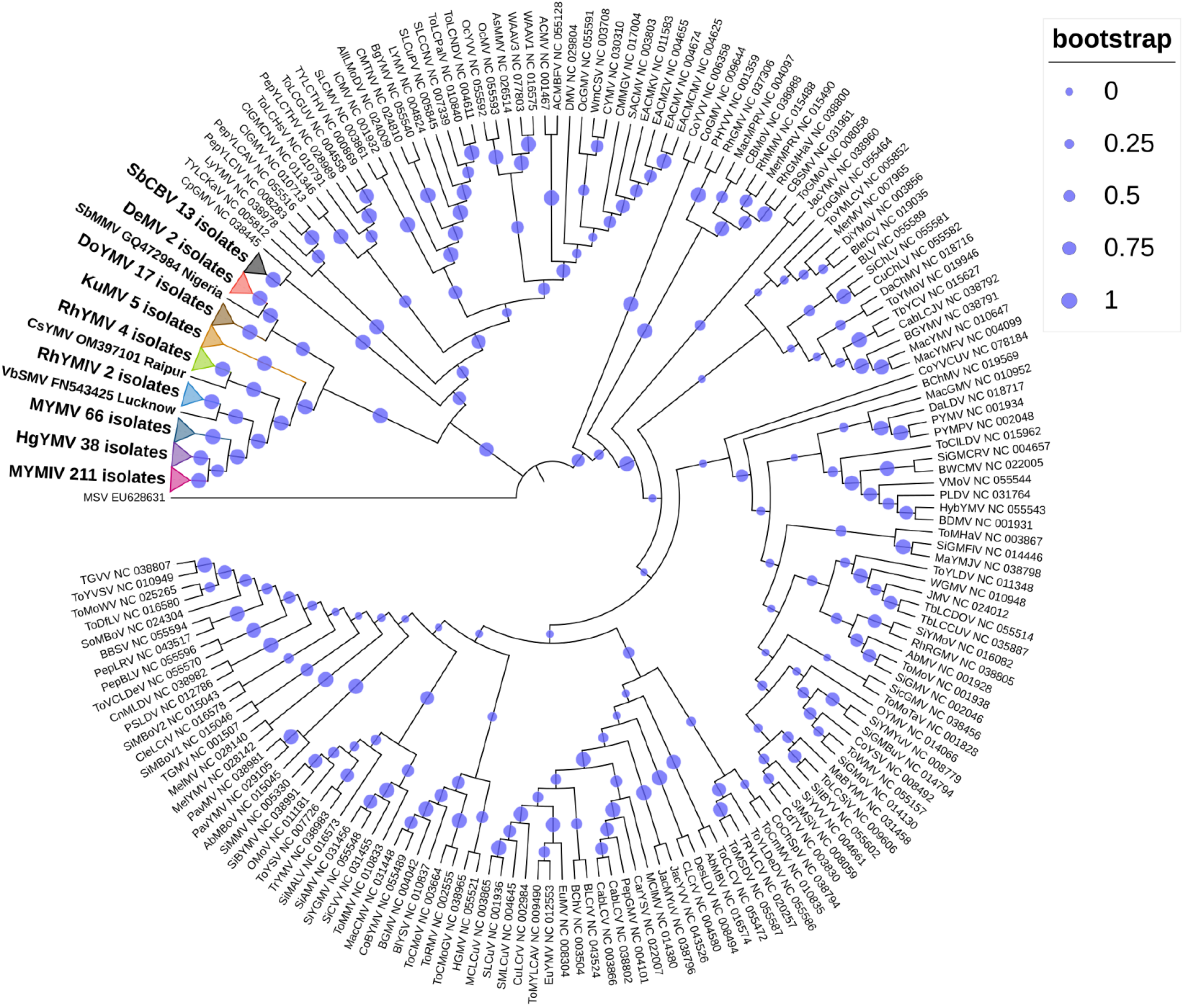
Phylogenetic tree derived from the DNA-A components of 530 begomoviruses. Legumoviruses considered in this study with only one isolate are shown with their corresponding species abbreviation, accession number, and location. Legumoviruses with multiple isolates are represented by collapsed clades, marked by colored triangles. Different colored triangles represents different species Other begomoviruses are shown in their abbreviated form followed by their RefSeq accession number, with detailed information provided in Supplementary Table S5. Bootstrap values, indicative of the reliability of each branch, are represented by the size of the blue circles at the nodes, with larger circles indicating higher confidence, as shown in the upper right.

**Figure 4:**
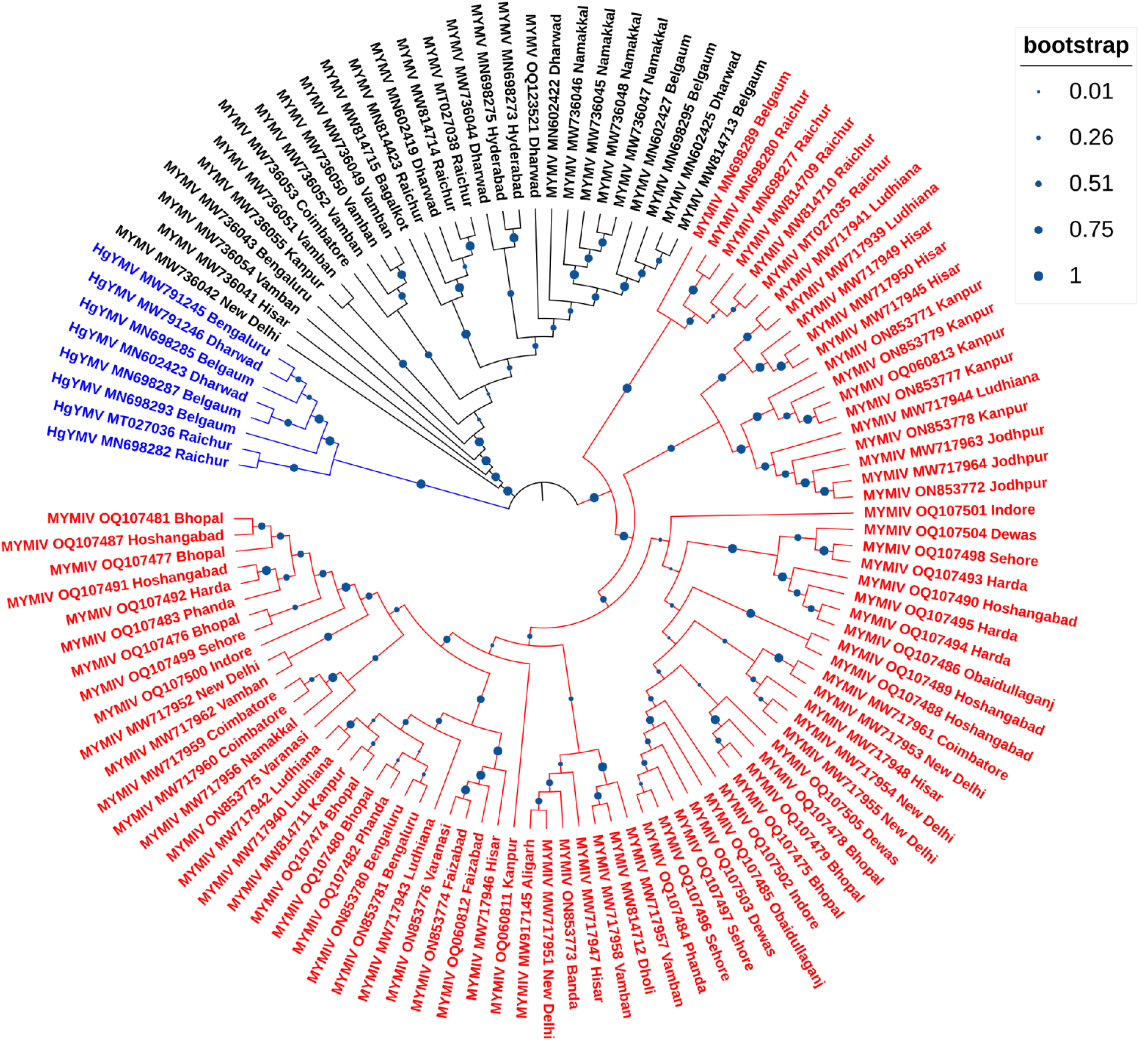
Phylogenetic tree derived from the DNA-A components of 117 full-length DNA-A sequences generated in this study comprising 81, 28 and 8 isolates of MYMIV, MYMV and HgYMV, respectively. Bootstrap values, indicative of the reliability of each branch, are represented by the size of the blue circles at the nodes, with larger circles indicating higher confidence, as shown in the upper right.

So far, a total of 445 begomoviruses have been cataloged by the ICTV, with 177 being bipartite. CsYMV had not been cataloged at the time of this study. Additionally, SbMMV, a legumovirus for which only the DNA-A component is known, and CpGMV, a monopartite begomovirus were also taken into consideration. Apart from legumoviruses, 167 bipartite species were considered, with only their NCBI RefSeq accessions of DNA-A and DNA-B components being taken into account (Supplementary Table S5). There were 361 full-length sequences of DNA-A and 303 full-length sequences of DNA-B components, representing isolates of all the 12 legumoviruses. Therefore, for the phylogenetic analysis, there were 530 DNA sequences of DNA-A and 471 DNA sequences of DNA-B, including MSV as an outgroup. The separate analysis for DNA-A sequences revealed two large clusters of OW and NW begomoviruses. The large cluster of OW was further observed to have two sub-clusters, one sub-cluster comprised 11 of the 12 legumoviruses considered in this study (Figure 3). SbCBV was observed in the other sub-cluster of OW begomovirus grouped with CpGMV. Both SbCBV and CpGMV have been reported from Nigeria. SbCBV could be considered as a connecting link, separating legumoviruses from other OW begomoviruses. Country-wise, distinct geographic lineages were evident among legumoviruses, particularly among MYMIV isolates. Isolates of MYMIV from Pakistan, Nepal, Oman, and Indonesia were generally found to cluster with isolates from their respective countries. Some MYMIV isolates from Hisar, Ludhiana, and New Delhi in the NWPZ of India were closely related to isolates from Pakistan. However, legumovirus isolates from India did not show differentiation based on location (Supplementary Figure S3). A separate phylogenetic analysis of 117 full-length DNA-A sequences generated in this study revealed clear species-level differentiation among MYMIV, MYMV, and HgYMV. Nevertheless, no location-based differentiation was observed among isolates of any of these three species (Figure 4). The phylogenetic analysis of DNA-B sequences resulted in one large cluster of NW begomoviruses and two distinct clusters (large and small) of OW begomoviruses. The large cluster of OW begomoviruses included a sub-cluster of legumoviruses considered in this study, along with a few other OW species, and a separate sub-cluster of additional OW species (Supplementary Figure S4). Isolates of SbCBV were observed separate from both of these sub-clusters within the large OW cluster, paired with CYMV (cotton yellow mosaic virus). The small cluster harbored the remaining OW begomoviruses. DNA-B components of several isolates of MYMIV and MYMV were highly similar and clustered together. Overall, the analysis showed that there was no clear distinction between the DNA-B components of MYMIV and MYMV.

### 3.6 Diversity estimation of legumoviruses

#### 3.6.1 National level

**Figure 5:**
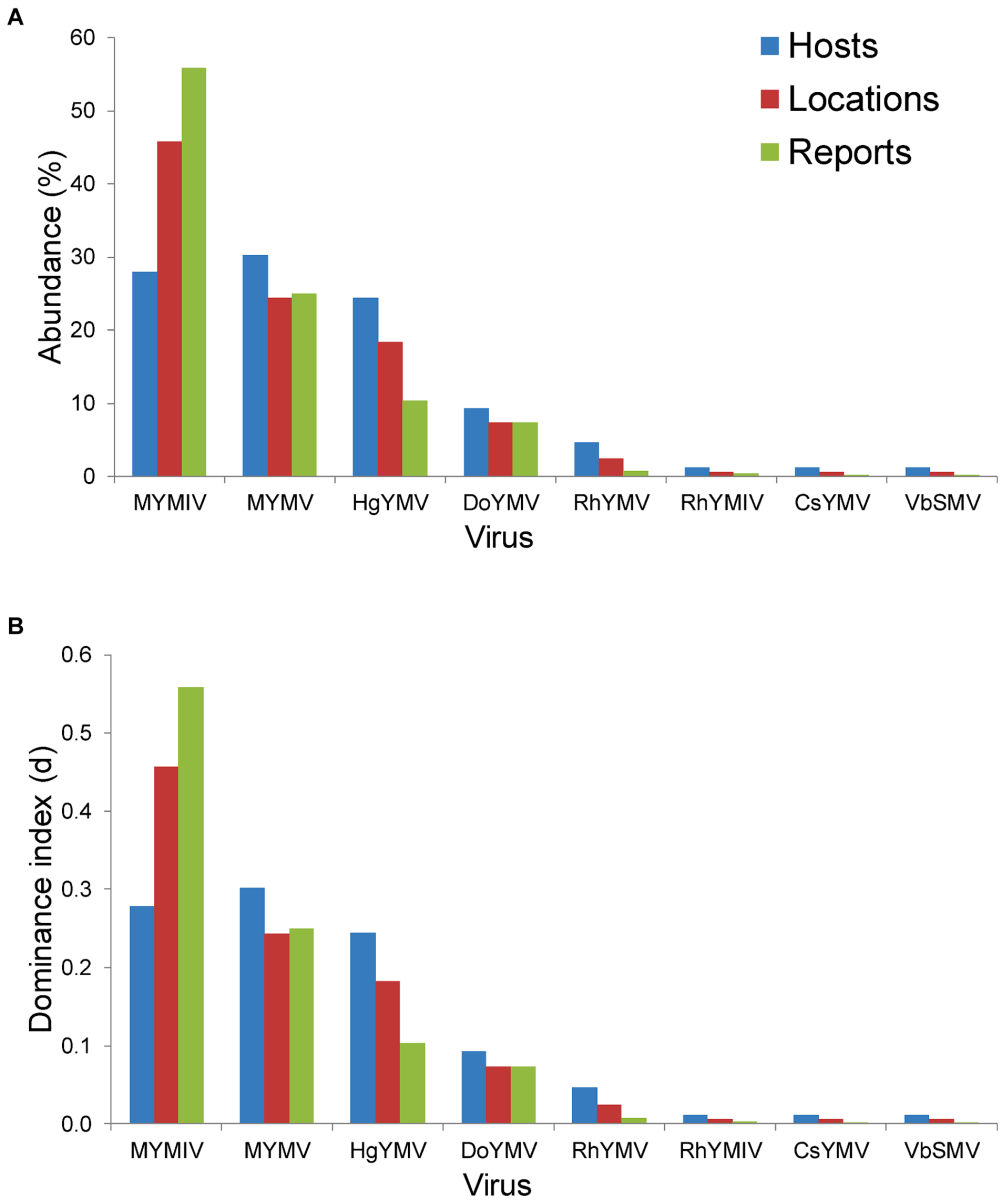
Histogram plot showing **(A)** per cent abundance of 8 virus namely MYMIV (mungbean yellow mosaic India virus), MYMV (mungbean yellow mosaic virus), HgYMV (horsegram yellow mosaic virus), DoYMV (dolichos yellow mosaic virus), RhYMV (Rhynchosia yellow mosaic virus), RhYMIV (Rhynchosia yellow mosaic India virus), CsYMV (*Cajanus scarabaeoides* yellow mosaic virus) and VbSMV (velvet bean severe mosaic virus); **(B)** Berger-Parker dominance index (d) of these 8 virus species; in terms of hosts, locations and total number of reports (or DPs) per virus species in India.

Across hosts, the MYMV was the most abundant (occurred on 30.2% of total hosts) and dominant (d = 0.3) in India, followed by MYMIV, HgYMV and others (Figure 5A). Whereas across locations and total DPs, MYMIV was the most abundant (55.9 and 45.7%, respectively) and dominant (0.56 and 0.46, respectively) virus, followed by MYMV, HgYMV and others (Figure 5B). Overall, the *H′* (1.58), ENS (4.86), *D_mg_* (1.57) and E (0.76) of virus species were higher across hosts followed by locations (*H′* = 1.39, ENS = 4.09, *D_mg_* = 1.37, and E = 0.67) and total DPs (*H′* = 1.18, ENS = 3.24, *D_mg_* = 1.1, and E = 0.57) (Table 3).

**Table 3:** Summary of diversity indices evaluated based on DNA-A-related information of legumoviruses at national and zonal level in India.

Abundance (%)									Berger-Parker’s Dominance Index (d)								*H’*	ENS	D_mg_	E
	Virus																			
Category	CsYMV	DoYMV	HgYMV	MYMIV	MYMV	RhYMIV	RhYMV	VbSMV	CsYMV	DoYMV	HgYMV	MYMIV	MYMV	RhYMIV	RhYMV	VbSMV				
**At National level**																				
Hosts	1.2	9.3	24.4	27.9	30.2	1.2	4.7	1.2	0.01	0.09	0.24	0.28	0.3	0.01	0.05	0.01	1.58	4.86	1.57	0.76
Locations	0.6	7.3	18.3	45.7	24.4	0.6	2.4	0.6	0.01	0.07	0.18	0.46	0.24	0.01	0.02	0.01	1.39	4.01	1.37	0.67
DPs	0.2	7.4	10.3	55.8	25.1	0.3	0.7	0.2	0	0.07	0.1	0.56	0.25	0	0.01	0	1.18	3.24	1.1	0.57
**South zone (SZ)**																				
Hosts	–	35.8	116.3	93.9	107.3	4.5	–	–	–	0.04	0.43	0.22	0.29	0.02	–	–	1.27	3.55	1.03	0.79
Locations	–	18.3	61	45.7	114.3	1.5	–	–	–	0.08	0.38	0.28	0.26	0.01	–	–	1.32	3.75	0.91	0.82
DPs	–	5.1	17.2	7.1	38.2	0.2	–	–	–	0.05	0.27	0.33	0.34	0.01	–	–	1.28	3.6	0.74	0.8
**Central zone (CZ)**																				
Hosts	5.3	5.3	–	63.2	15.8	–	10.5	–	0.05	0.05	–	0.63	0.16	–	0.11	–	1.13	3.09	1.36	0.7
Locations	3.1	6.3	–	68.8	15.6	–	6.3	–	0.03	0.06	–	0.69	0.16	–	0.06	–	1.00	2.73	1.15	0.62
DPs	0.8	1.6	–	91	4.9	–	1.6	–	0.01	0.02	–	0.91	0.05	–	0.02	–	0.41	1.5	0.83	0.25
**North east plain zone (NEPZ)**																				
Hosts	–	26.9	–	30.8	30.8	–	7.7	3.8	–	0.27	–	0.31	0.31	–	0.08	0.04	1.4	4.06	1.23	0.87
Locations	–	10.3	–	69	10.3	–	6.9	3.4	–	0.1	–	0.69	0.1	–	0.07	0.03	1.03	2.79	1.19	0.64
DPs	–	20.6	–	59.5	17.5	–	1.6	0.8	–	0.21	–	0.6	0.17	–	0.02	0.01	1.04	2.84	0.83	0.65
**North west plain zone (NWPZ)**																				
Hosts	–	4.3	–	39.1	56.5	–	–	–	–	0.04	–	0.39	0.57	–	–	–	0.84	2.31	0.64	0.52
Locations	–	5.6	–	33.3	61.1	–	–	–	–	0.06	–	0.33	0.61	–	–	–	0.81	2.25	0.69	0.5
DPs	–	3.8	–	53.8	42.3	–	–	–	–	0.04	–	0.54	0.42	–	–	–	0.84	2.31	0.43	0.52

(−) indicates the absence of virus in a zone; DPs, Total data points; *H*′, Shannon-Weiner Diversity index; ENS, Effective Number of Species; D_mg_, Species Richness; E, Species Evenness.

#### 3.6.2 Zonal level

At the zonal level, there were five virus species, each recorded from SZ, CZ and NEPZ, and three virus species from NWPZ. However, there was only one species occurring in the NHZ. The diversity indices were determined for the first four zones, where more than two species occurred. In the SZ, HgYMV was the most abundant and dominant species across hosts (42.9% and d = 0.43) and locations (37.5% and d = 0.38) followed by MYMV, MYMIV, DoYMV and RhYMV. However, across total DPs, both MYMV and MYMIV were equally abundant and dominant (33–33.5% and d = 0.33) followed by HgYMV, DoYMV and RhYMV. In the CZ, MYMIV was the most abundant and dominant species across hosts, locations and total reports (63.2–91% and d = 0.63–0.91) followed by the other three species (5–15% and d = 0.05–0.16). In the NEPZ, both MYMIV and MYMV were equally abundant and dominant species (30.85% and d = 0.31), however, across locations and total DPs, MYMIV was the most abundant and dominant (59.5–69% and d = 0.60–0.69) species. Interestingly, when total DPs were considered, DoYMV followed MYMIV occurring in nearly 21% of the DPs followed by MYMV, which occurred in 17% of the DPs. In the NWPZ, MYMV was the most abundant and dominant species across hosts and locations (56.5–61.1% and d = 0.57–0.61), however, it was MYMIV across the total DPs (53.8% and d = 42.3) (Table 3).

The diversity indices varied between different zones of India. Overall, NEPZ recorded a higher Shannon-Weiner diversity index (*H′* = 1.4, 1.03, and 1.04, respectively across hosts, locations and total DPs) and Effective Number Species (ENS = 4.06, 2.79 and 2.84), followed by SZ (*H′* = 1.27, 1.32 and 1.28; ENS = 3.55, 3.75 and 3.6), CZ (*H′* = 1.13, 1.0 and 0.41; ENS = 3.09, 2.73 and 1.5) and NWPZ (*H′* = 0.84, 0.81 and 0.84; ENS = 2.31, 2.25 and 2.31). Further, CZ (*D_mg_* = 1.36, 1.15 and 0.83) and NEPZ (*D_mg_ =* 1.23, 1.19 and 0.83) recorded similar species richness indexes; respectively across hosts, locations and total DPs. This was followed by SZ (*D_mg_* = 1.03, 0.9 and 0.74) and NWPZ (*D_mg_* = 0.64, 0.69 and 0.43). However, with respect to species evenness (E), SZ recorded the highest evenness (E = 0.79, 0.82 and 0.81), respectively, across hosts, locations and total DPs. This was followed by NEPZ (E = 0.84, 0.64 and 0.65), CZ (E = 0.7, 0.62 and 0.25) and NWPZ (E = 0.52, 0.5 and 0.52) (Table 3; Supplementary Table S6).

## 4 Discussion

As far as the research reports on yellow mosaic disease (YMD) in pulse crops are concerned, the maximum focus has been on mungbean (54%) followed by urdbean (25%) and soybean. The YMD in these 3 crops was observed to be caused mainly by MYMIV in all the five agro-climatic zones followed by MYMV, which is predominant in the South Zone (SZ). Urdbean contributing about 14% of the total pulses granary of India (Project Coordinator Report, 2022) is cultivated in northern parts of India mainly as a *Kharif* and spring season crop. However, in the central and southern parts of India, it is mainly grown as a *Kharif* or rainy season crop. YMD in urdbean grown in these regions is mainly caused by MYMIV and MYMV with yield losses of up to 100% (Sen Gupta et al., 2023). Mungbean is mainly grown in different parts of India in all three seasons, namely *Kharif*, *Rabi*, and *Zaid* (Jakhar et al., 2016). YMD is also known to affect several other leguminous pulse crops such as limabean, mothbean, pigeonpea, French bean, dolichos, horsegram, cowpea, and soybean (Mishra et al., 2020). MYMIV is known to be prevalent in the northern and central parts of India (Kumar et al., 2014a), whereas MYMV is prevalent in the southern region of India (Usharani et al., 2004; Kumar et al., 2014b).

PCR-based detection of legumoviruses using species-specific primers has been demonstrated many times for their identification and characterization (Naimuddin et al., 2011c, 2016; Agnihotri et al., 2019; Akram et al., 2020, 2022). In this study, we have processed the samples utilizing species-specific primers of four legumoviruses collected from five different agro-climatic zones for the identification and complete genome characterization. This study provided invaluable insight into the prevalence of YMD-causing legumoviruses in India. While utmost care was taken to represent all zones, the NHZ represents comparatively few samples, primarily because of the very little area under cultivation of *Vigna* pulses and also COVID-related logistic limitations during the survey period. Among all the samples, MYMIV was detected in the majority, followed by MYMV, HgYMV, and DoYMV. Given their importance, these four legumoviruses in agriculture have been the focus of attention and therefore of primary research. It is known that these legumoviruses can cause substantial financial losses to the farmers in areas where they are most prevalent. Numerous studies have been carried out to understand molecular biology, transmission, host interactions, epidemiology, and the management techniques of these viruses (Naimuddin et al., 2016; Mishra et al., 2020). Interestingly, MYMIV has been reported from numerous locations, indicating its widespread distribution. HgYMV has been primarily observed in the southern parts of India, suggesting a more localized presence. A similar observation of another virus, tomato leaf curl Palampur virus (ToLCPalV) was reported to be localized to Northern India with reasons attributed to factors such as vector dynamics, climate suitability, host range, human activity, natural barriers and evolutionary processes (Nayaka et al., 2023).

In the phylogenetic analysis of DNA-A sequences, excluding SbCBV, the remaining 11 legumoviruses considered in this study formed a distinct sub-cluster within the single large cluster of OW begomoviruses, consistent with a previous report (Naimuddin et al., 2016). The sub-cluster of legumoviruses was observed to be partitioned from the other OW and NW bipartite begomoviruses by SbCBV observed closely grouped with CpGMV revealing geographically defined lineages similar to the observations of a previous study, which reported SbCBV as a bipartite ‘African legumovirus’ (Alabi et al., 2010). In a previous study, before the identification of CsYMV (Dokka et al., 2023), 11 species of begomoviruses were suggested to be the members of legumoviruses based on their phylogenetic analysis (Naimuddin et al., 2016). SbCBV lacks the gene AV2 usually present in the OW begomoviruses (Alabi et al., 2010). The absence of AV2 in SbCBV and its similarity with CpGMV makes it a likely connecting link between the sub-clusters of other OW begomoviruses and legumoviruses. In a separate study, certain legume-infecting begomoviruses originating from the Old World, particularly from the Americas, were identified as distinct from others of the same category in phylogenetic analysis forming a separate cluster (Fauquet et al., 2008). Isolates of MYMIV and MYMV considered in this study cumulatively shared about 55% sequence similarity, which supports their classification as distinct species in the phylogenetic analysis of DNA-A components. Similar distinction between MYMIV and MYMV was not observed in case of phylogenetic analysis of DNA-B components where several isolates of MYMIV and MYMV were grouped with each other. This observation could be the outcome of reassortment events involving DNA-B components occurred during mixed infection in a common host (Balaji et al., 2004; John et al., 2008; Rouhibakhsh et al., 2008). Also, having >75% similarity in the common region and common hosts of these two species, DNA-A of one species could capture DNA-B of other species and vice versa (Haq et al., 2011). This genetic exchange could blur the phylogenetic boundaries between these viruses (MYMIV and MYMV) in certain analyses.

Apart from leguminous hosts, including crops and weeds, some legumoviruses are reported to infect non-leguminous weed hosts (Naimuddin et al., 2014; Bhanu et al., 2015; Marabi et al., 2017, 2021; Chowdary et al., 2022). Whiteflies, being polyphagous insects, are not limited by plant type and can transmit begomoviruses across various hosts within the *Fabaceae* family and plants from other families. Reports of DoYMV (this study) and HgYMV (unpublished) infecting weed hosts based on characterized partial sequence are available in the public database (Supplementary Table S3). Reports on several weed hosts growing near urdbean fields were found infected with MYMIV from Andhra Pradesh (SZ) (Chowdary et al., 2022). No direct correlation between symptoms of YMD and the presence of the virus, or vice versa, was observed in a study conducted for the detection of MYMIV and MYMV in weeds. Both MYMIV and MYMV can survive as latent, showing no symptoms in weed hosts (Bhanu et al., 2015). In such a way, they act as the source of primary inoculum for the main crops (Naimuddin et al., 2016). There are reports that these viruses have been detected in non-symptomatic samples of mungbean, urdbean and *Capsicum* (Biswas and Varma, 2000, 2001; Biswas et al., 2005; Polston et al., 2006) and can serve as a source of primary inoculum for their further spread to the main crops.

An effort was undertaken to document the distribution of MYMIV, MYMV, DoYMV, and HgYMV relying solely on published reports, but it provided limited information on the distribution of legumoviruses in India (Sahu et al., 2021). In contrast, this study is based on a survey, testing 174 samples from 32 locations across five agro-climatic zones and confirming the identity of viruses causing yellow mosaic disease (YMD) through full genome characterization of 117 DNA-A components representing three legumoviruses. This approach aimed to eliminate any ambiguity that might arise solely from PCR-based detection. Additionally, we included 464 accessions specific to the DNA-A component, representing eight legumoviruses detected across India. This comprehensive approach allowed us to accurately depict the geographical distribution of these viruses. Another study employed a similar approach to understand the geographical distribution of tomato-infecting begomoviruses in cucurbit crops in India (Nayaka et al., 2023). However, this study solely relied on PCR-based detection of begomovirus using species-specific primers. Notably, amplicons were not validated by sequencing (Nayaka et al., 2023). Several other studies attempted to generate information on the distribution of Begomoviruses associated with cassava (Anuradha and Karumannil, 2022), tomato (Saha et al., 2014), sunflower (Vindyashree et al., 2016), and mungbean/urdbean (Srinivasaraghavan et al., 2021; Dhobale et al., 2023).

Assessment of diversity indices helps us to understand the commonness and rarity of any living organisms on this earth. Several studies used various diversity analyses to understand the distribution of living organisms in a niche, community, or habitat. For instance, the diversity indices were successfully utilized to understand the plants (Fattorini et al., 2016), insects (Revanasidda and Belavadi, 2019; Aidbhavi et al., 2023) and microbial (Kim et al., 2017) diversities. Measuring the diversity of viruses in various environments continues to pose a challenge compared to measuring the diversity of other microbial communities. Nevertheless, the fundamental diversity indices, such as the Shannon-Weiner index, richness, and evenness index, provide valuable insights when data from multiple communities or habitats are compiled (Herath et al., 2017). Considering the quantum of data collected in this study, efforts were made to understand the occurrence of virus species in the world, India and zones within India. In the present study, among the 12 virus species reported globally, there were only 4.4, 4.73 and 3.09 effective number of species, respectively across hosts, locations and total DPs (reports). This might be attributed to the dominance of three virus species (MYMV, MYMIV and HgYMV). The diversity indices such as *H′*, D_mg_ and E were higher across hosts and locations than across total DPs. This was because of the predominant occurrence of MYMIV in nearly 411 (57%) of the total 717 DPs worldwide. Interestingly, India contributes nearly 79% of the total MYMIV DPs worldwide, indicating the dominance level of MYMIV in India regarding total reports made within the country.

At the national level, though there are eight viruses reported from India, the ENS indicates that there are only 4.86, 4.01 and 3.24 virus species present, respectively, across hosts, locations and total DPs. This might be attributed to the higher abundance and dominance of three species (MYMV, MYMIV and HgYMV) which contribute to nearly 83, 88 and 91% of the total virus reports across hosts, locations and total DPs, respectively. Similarly, higher *H′*, D_mg_ and E across hosts indicate the increased occurrence of other virus species (other than the major species, i.e., MYMV, MYMIV and HgYMV) across hosts when compared to locations followed by total DPs. Overall, the diversity indices reveal that the virus species MYMV is dominant globally, whereas MYMIV is dominant in India.

Across five agro-climatic zones in India, the MYMIV species was the most abundant and dominant across different zones when total DPs were considered. In SZ, though the HgYMV was the most abundant species across hosts and locations, but when the total DPs were considered, MYMV and MYMIV were the most abundant and dominant. Notably, several samples collected from the SZ in this study exhibited mixed infections of MYMIV, MYMV and HgYMV as compared to the other zones. This could be attributed to the climatic conditions of the SZ, which provide an ideal environment for whiteflies to flourish during the summer and *Kharif* seasons (Perring et al., 2018). In both CZ and NEPZ, MYMIV was the predominant species. In NWPZ, although MYMV was predominant across hosts and locations, it was MYMIV across total DPs. Even though five species occurred across three zones; the ENS value indicated that the actual number of virus species was 1.5–4. This was because of the predominant occurrence of MYMIV and MYMV. The higher species richness index and lower evenness in CZ might be attributed to the predominant distribution of single virus species (MYMIV) in more than 60% of the hosts, locations and total DPs. Although SZ recorded lower *H′* and ENS than NEPZ, it has recorded higher E values that might be attributed to the distribution of three out of five viruses in more than 20% of hosts, locations and total DPs. Susi et al. (2019) attempted to understand the variations in five species of viruses among populations using the Shannon-Weiner (*H′*) index and found that the *H′* value ranged between 0.001 and 0.005 which is much lesser than what we determined in the present study. Williamson et al. (2005) studied the abundance and diversity of autochthonous viruses across six Delaware soil types representing forest, agricultural, and plain habitats. The diversity indices revealed that the forest soils harbored more viral diversity than the other two soil types.

Overall, this study could have several significant implications for the researchers working on legume crops. For example, it could help to understand the spread of legumoviruses and their geographic distribution. The information on regions/zones having a predominance of certain legumovirus could help researchers estimate the crop damage, thereby making it easy to take decisions on management practices including deployment of resistant varieties or pest control measures to mitigate the impact of the virus on crop yield. The knowledge of legumovirus distribution could further aid in improving breeding programs, directing the efforts of breeders towards the development of resistant varieties. In addition, the researchers can prioritize specific areas and regions with prevailing multiple virus species for an effective germplasm screening program and subsequently identify robust and stable sources of resistance to deploy them in nation-wide breeding efforts. Policymakers can also utilize the map to revise pulse/legume crop policies and allocate resources accordingly. Targeted interventions, such as providing support to the farmers in high-risk areas or investing in research and extension services, can help mitigate the impact of legumoviruses on crop production. Collaborative efforts at both national and international levels, with a unified focus, can further enhance our knowledge of virus prevalence and distribution in previously unexplored areas where legume crops are cultivated. Additionally, these collaborations can aid in predicting the emergence of recombinant species.

## Data Availability

The datasets presented in this study can be found in online repositories. The names of the repository/repositories and accession number(s) can be found in the article/Supplementary material.

## Footnotes

^1^https://blast.ncbi.nlm.nih.gov/Blast.cgi

^2^https://www.ncbi.nlm.nih.gov/genbank

^3^https://www.ncbi.nlm.nih.gov/orffinder

^4^https://www.ncbi.nlm.nih.gov/nuccore

^5^https://www.google.com/maps

^6^https://www.latlong.net/

## Supplementary material

SUPPLEMENTARY FIGURE S1 An outline map of India displaying the geographical distribution of legumovirus species, with colored spots indicating specific locations for detecting; **(A)** mungbean yellow mosaic India virus, **(B)** mungbean yellow mosaic virus, **(C)** dolichos yellow mosaic virus, **(D)** horsegram yellow mosaic virus, Rhynchosia yellow mosaic virus, Rhynchosia yellow mosaic India virus and Cajanus scarabaeoides yellow mosaic virus.

SUPPLEMENTARY FIGURE S2 Pie charts illustrating the host range and the number of reports per host for the detection of mungbean yellow mosaic India virus (MYMIV), mungbean yellow mosaic virus (MYMV), dolichos yellow mosaic virus (DoYMV), and horsegram yellow mosaic virus (HgYMV) from India

SUPPLEMENTARY FIGURE S3 Phylogenetic tree derived from the DNA-A components of 530 begomoviruses. Isolates of all the 12 legumoviruses considered in this study are shown with their corresponding species abbreviation, accession number, and location. Isolates reported from countries other than India are highlighted with different colors. Other begomoviruses are shown as collapsed clade represented by grey triangles. Bootstrap values, indicative of the reliability of each branch, are represented by the size of the blue circles at the nodes, with larger circles indicating higher confidence, as shown in the upper right.

SUPPLEMENTARY FIGURE S4 Phylogenetic tree derived from the DNA-B components of 471 begomoviruses. Legumoviruses considered in this study with only one isolate are shown with their corresponding abbreviation, accession number, and location. Legumoviruses with multiple isolates are represented by collapsed clades, marked by colored triangles. Different colored triangles represents different species. Other begomoviruses are shown in their abbreviated form followed by their RefSeq accession number, with detailed information provided in Supplementary Table S5. Bootstrap values, indicative of the reliability of each branch, are represented by the size of the blue circles at the nodes, with larger circles indicating higher confidence, as shown in the upper right.
