# Supplementary figures and images for: Exploring distribution and genomic diversity of begomoviruses associated with yellow mosaic disease of legume crops from India, highlighting the dominance of mungbean yellow mosaic India virus
Source: Front Microbiol. 2024 Aug 27;15:1451986. doi: 10.3389/fmicb.2024.1451986 (PMC11385007; doi:10.3389/fmicb.2024.1451986)

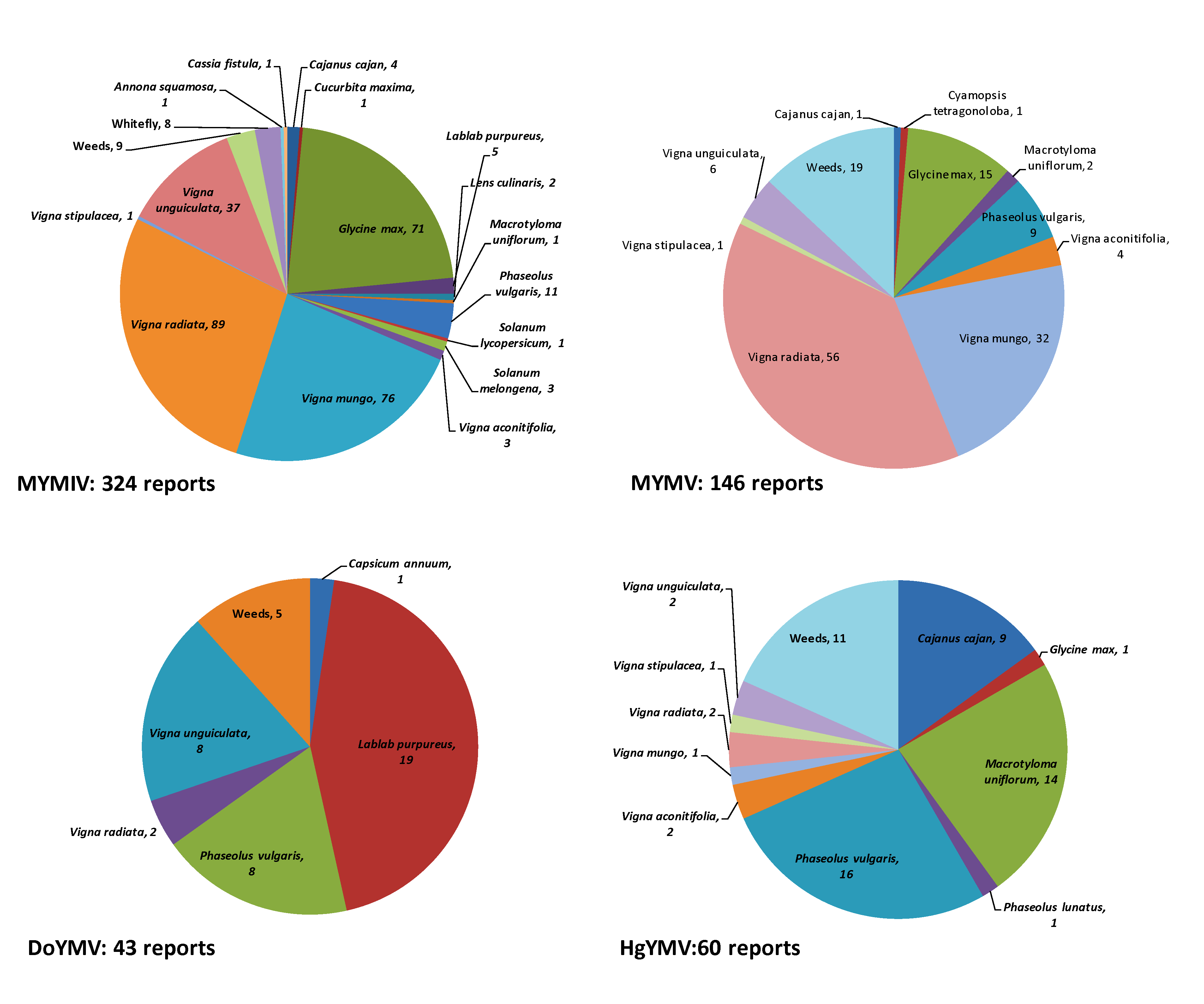

Supplement: SUPPLEMENTARY FIGURE S2 — Pie charts illustrating the host range and the number of reports per host for the detection of mungbean yellow mosaic India virus (MYMIV), mungbean yellow mosaic virus (MYMV), dolichos yellow mosaic virus (DoYMV), and horsegram yellow mosaic virus (HgYMV) from India [file Image_2.TIFF]

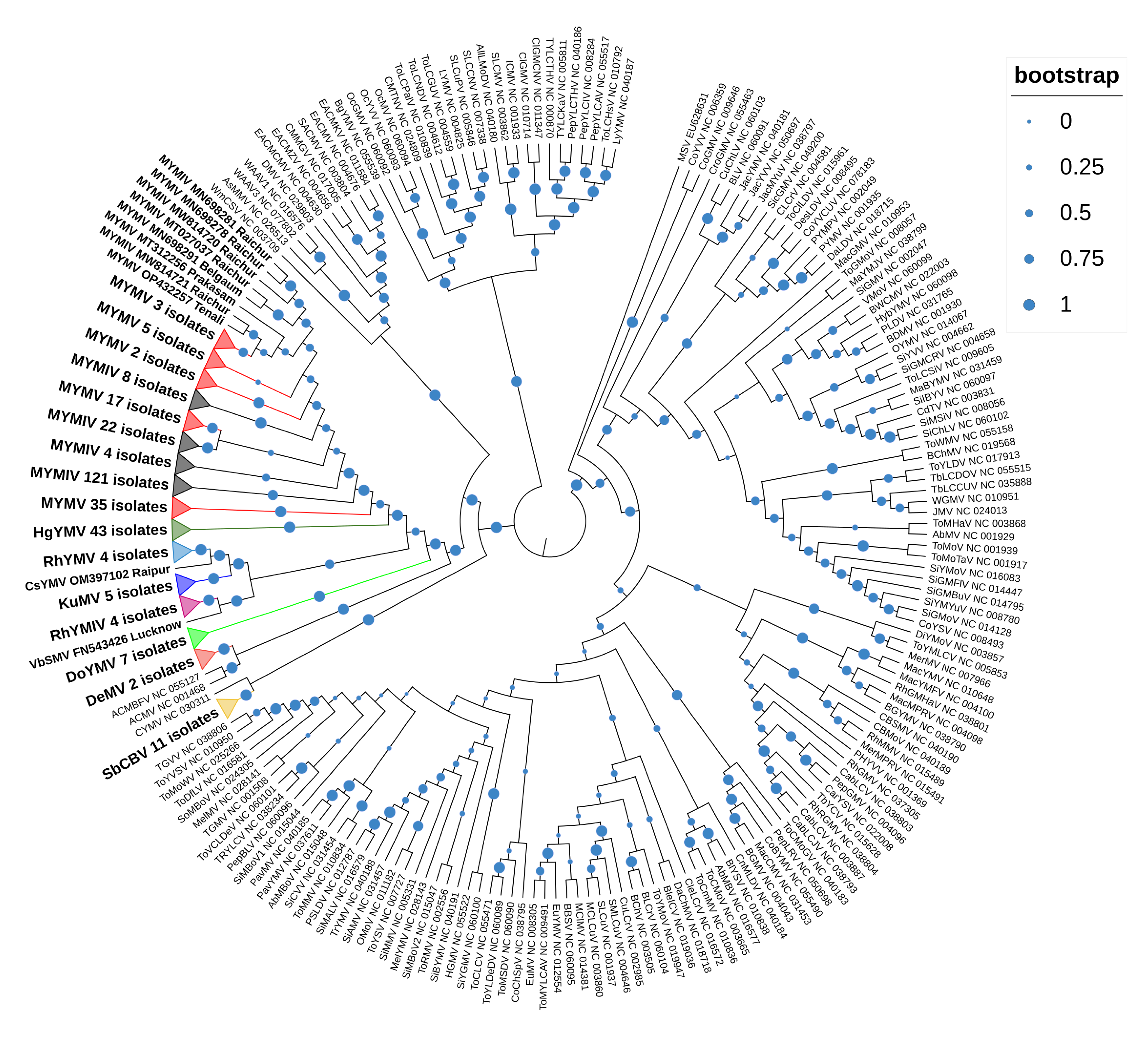

Supplement: SUPPLEMENTARY FIGURE S4 — Phylogenetic tree derived from the DNA-B components of 471 begomoviruses. Legumoviruses considered in this study with only one isolate are shown with their corresponding abbreviation, accession number, and location. Legumoviruses with multiple isolates are represented by collapsed clades, marked by colored triangles. Different colored triangles represents different species. Other begomoviruses are shown in their abbreviated form followed by their RefSeq accession number, with detailed information provided in Supplementary Table S5. Bootstrap values, indicative of the reliability of each branch, are represented by the size of the blue circles at the nodes, with larger circles indicating higher confidence, as shown in the upper right. [file Image_4.TIFF]
