# Supplementary material for: Exploring distribution and genomic diversity of begomoviruses associated with yellow mosaic disease of legume crops from India, highlighting the dominance of mungbean yellow mosaic India virus
Source: Front Microbiol. 2024 Aug 27;15:1451986. doi: 10.3389/fmicb.2024.1451986 (PMC11385007; doi:10.3389/fmicb.2024.1451986)
Supplement: Supplementary file 5 [file Table_1.DOCX]

**Supplementary Table S1** Details of primers used in PCR for the detection of legumoviruses

| **YMD-causing virus/component** | **Gene** | **Name** | **Sequence (5′ to 3′)** | **Annealing temperature (°C)** | **Amplicon size**  **(base pairs)** |
| --- | --- | --- | --- | --- | --- |
| *Mungbean yellow mosaic India virus/* DNA-A | Coat protein | MYMIV-CP14 (F)  MYMIV-CP14 (R) | 5’-TGCATGGATTCCGGTGCATG-3’  5’-GGTGGAGGATGTAAGCTTTAC-3’ | 56 | ~900 |
| *Mungbean yellow mosaic virus/* DNA-A | Coat protein | MYMV-CP14 (F)  MYMV-CP14 (R) | 5’-GGCAGCCATGTGGGATCCA-3’  5’-GGCGTCATTAGCATAGGCAAT-3’ | 56 | ~1000 |
| *Dolichos yellow mosaic virus/* DNA-A | Coat protein | DoYMV-CP14 (F)  DoYMV-CP14 (R) | 5’-TTAGCTGTGAAATTTGTGCAGG-3’  5’-TCTGGGCGGATCTTGAATGTA-3’ | 56 | ~1050 |
| *Horsegram yellow mosaic virus/* DNA-A | Coat protein | HgYMV-CP14 (F)  HgYMV-CP14 (R) | 5’-GTATGCTTGCAATTAAGTACTTG-3’  5’-ACATCGTCCAATGGGAGCAG-3’ | 56 | ~1180 |
