# Supplementary material for: Exploring distribution and genomic diversity of begomoviruses associated with yellow mosaic disease of legume crops from India, highlighting the dominance of mungbean yellow mosaic India virus
Source: Front Microbiol. 2024 Aug 27;15:1451986. doi: 10.3389/fmicb.2024.1451986 (PMC11385007; doi:10.3389/fmicb.2024.1451986)
Supplement: Supplementary file 8 [file Table_4.DOCX]

**Supplementary Table S4** List of locations from India with 581 reports on detection of eight legumoviruses in different hosts.

| **Sl.** | **Location** | **Host** | **Legumovirus*** | | | | | | | | |
| --- | --- | --- | --- | --- | --- | --- | --- | --- | --- | --- | --- |
|  |  |  | **Reports** | **MYMIV** | **MYMV** | **DoYMV** | **HgYMV** | **RhYMV** | **RhYMIV** | **CsYMV** | **VbSMV** |
|  | Akola, Maharashtra, India | *Phaseolus vulgaris* | 1 | **+** | **-** | **-** | **-** | **-** | **-** | **-** | **-** |
|  |  | *Vigna radiata* | 1 | **+** | **-** | **-** | **-** | **-** | **-** | **-** | **-** |
|  | Aligarh, Uttar Pradesh, India | *Vigna mungo* | 1 | **+** | **-** | **-** | **-** | **-** | **-** | **-** | **-** |
|  | Anand, Gujarat, India | *Bemisia tabaci* | 4 | **+** | **-** | **-** | **-** | **-** | **-** | **-** | **-** |
|  |  | *Vigna aconitifolia* | 1 | **+** | **-** | **-** | **-** | **-** | **-** | **-** | **-** |
|  |  | *Vigna mungo* | 1 | **+** | **-** | **-** | **-** | **-** | **-** | **-** | **-** |
|  |  | *Vigna radiata* | 2 | **+** | **-** | **-** | **-** | **-** | **-** | **-** | **-** |
|  | Anantapuram, Andhra Pradesh, India | *Cajanus cajan* | 3 | **-** | **-** | **-** | **+** | **-** | **-** | **-** | **-** |
|  |  | *Macrotyloma uniflorum* | 1 | **-** | **-** | **-** | **+** | **-** | **-** | **-** | **-** |
|  | Annur, Tamilnadu, India | *Vigna radiata* | 1 | **-** | **+** | **-** | **-** | **-** | **-** | **-** | **-** |
|  | Aurangabad, Maharashtra, India | *Glycine max* | 1 | **+** | **-** | **-** | **-** | **-** | **-** | **-** | **-** |
|  | Bagalkot, Karnataka, India | *Vigna radiata* | 1 | **-** | **+** | **-** | **-** | **-** | **-** | **-** | **-** |
|  | Bagepalli, Karnataka, India | *Phaseolus vulgaris* | 1 | **-** | **-** | **-** | **+** | **-** | **-** | **-** | **-** |
|  | Banda, Uttar Pradesh, India | *Vigna radiata* | 1 | **+** | **-** | **-** | **-** | **-** | **-** | **-** | **-** |
|  | Bangarapete, Karnataka, India | *Phaseolus vulgaris* | 1 | **-** | **-** | **-** | **+** | **-** | **-** | **-** | **-** |
|  | Bapatla, Andhra Pradesh, India | *Vigna mungo* | 1 | **+** | **-** | **-** | **-** | **-** | **-** | **-** | **-** |
|  | Bardhaman, West Bengal , India | *Vigna mungo* | 2 | **+** | **-** | **-** | **-** | **-** | **-** | **-** | **-** |
|  | Bareilly, Uttar Pradesh, India | *Vigna unguiculata* | 2 | **+** | **-** | **-** | **-** | **-** | **-** | **-** | **-** |
|  | Baronda, Chhattisgarh, India | *Vigna radiata* | 2 | **+** | **-** | **-** | **-** | **-** | **-** | **-** | **-** |
|  | Bathinda, Punjab, India | *Vigna radiata* | 4 | **-** | **+** | **-** | **-** | **-** | **-** | **-** | **-** |
|  | Begusarai, Bihar, India | *Vigna radiata* | 1 | **-** | **+** | **-** | **-** | **-** | **-** | **-** | **-** |
|  | Belgaum, Karnataka, India | *Vigna aconitifolia* | 5 | **+** | **+** | **-** | **+** | **-** | **-** | **-** | **-** |
|  |  | *Vigna radiata* | 1 | **-** | **+** | **-** | **-** | **-** | **-** | **-** | **-** |
|  |  | *Vigna unguiculata* | 1 | **-** | **-** | **-** | **+** | **-** | **-** | **-** | **-** |
|  | Bengaluru, Karnataka, India | *Ageratum conyzoides* | 1 | **-** | **-** | **-** | **+** | **-** | **-** | **-** | **-** |
|  |  | *Euphorbia heterophylla* | 1 | **-** | **-** | **-** | **+** | **-** | **-** | **-** | **-** |
|  |  | *Glycine max* | 1 | **-** | **-** | **-** | **+** | **-** | **-** | **-** | **-** |
|  |  | *Lablab purpureus* | 2 | **-** | **-** | **+** | **-** | **-** | **-** | **-** | **-** |
|  |  | *Macrotyloma uniflorum* | 1 | **-** | **-** | **-** | **+** | **-** | **-** | **-** | **-** |
|  |  | *Phaseolus lunatus* | 1 | **-** | **-** | **-** | **+** | **-** | **-** | **-** | **-** |
|  |  | *Phaseolus vulgaris* | 5 | **-** | **+** | **-** | **+** | **-** | **-** | **-** | **-** |
|  |  | *Senna tora* | 1 | **-** | **-** | **-** | **+** | **-** | **-** | **-** | **-** |
|  |  | *Vigna mungo* | 1 | **-** | **+** | **-** | **-** | **-** | **-** | **-** | **-** |
|  |  | *Vigna radiata* | 2 | **-** | **+** | **-** | **-** | **-** | **-** | **-** | **-** |
|  |  | *Vigna unguiculata* | 3 | **+** | **-** | **-** | **+** | **-** | **-** | **-** | **-** |
|  | Bhagalpur, Bihar, India | *Cajanus cajan* | 1 | **+** | **-** | **-** | **-** | **-** | **-** | **-** | **-** |
|  |  | *Lens culinaris* | 2 | **+** | **-** | **-** | **-** | **-** | **-** | **-** | **-** |
|  | Bhopal, Madhya Pradesh, India | *Glycine max* | 2 | **+** | **-** | **-** | **-** | **-** | **-** | **-** | **-** |
|  |  | *Vigna radiata* | 8 | **+** | **-** | **-** | **-** | **-** | **-** | **-** | **-** |
|  | Bhubaneshwar, Odisha, India | *Vigna radiata* | 1 | **+** | **-** | **-** | **-** | **-** | **-** | **-** | **-** |
|  | Chamaluru, Andhra Pradesh, India | *Macrotyloma uniflorum* | 1 | **-** | **-** | **-** | **+** | **-** | **-** | **-** | **-** |
|  | Chamarajanagar, Karnataka, India | *Croton bonplandianus* | 1 | **-** | **+** | **-** | **-** | **-** | **-** | **-** | **-** |
|  |  | *Cynodon dactylon* | 1 | **-** | **-** | **-** | **+** | **-** | **-** | **-** | **-** |
|  |  | *Macroptilium atropurpureum* | 1 | **-** | **+** | **-** | **-** | **-** | **-** | **-** | **-** |
|  |  | *Macrotyloma uniflorum* | 1 | **-** | **+** | **-** | **-** | **-** | **-** | **-** | **-** |
|  |  | *Parthenium hysterophorus* | 1 | **-** | **-** | **-** | **+** | **-** | **-** | **-** | **-** |
|  |  | *Vigna mungo* | 1 | **-** | **+** | **-** | **-** | **-** | **-** | **-** | **-** |
|  |  | *Vigna radiata* | 3 | **-** | **+** | **-** | **+** | **-** | **-** | **-** | **-** |
|  | Chikkaballapura, Karnataka, India | *Acanthospermum hispidum* | 1 | **-** | **-** | **-** | **+** | **-** | **-** | **-** | **-** |
|  |  | *Alternanthera sessilis* | 1 | **-** | **+** | **-** | **-** | **-** | **-** | **-** | **-** |
|  |  | *Commelina benghalensis* | 1 | **-** | **+** | **-** | **-** | **-** | **-** | **-** | **-** |
|  |  | *Phaseolus vulgaris* | 1 | **-** | **-** | **-** | **+** | **-** | **-** | **-** | **-** |
|  |  | *Spermacoce articularis* | 1 | **-** | **-** | **-** | **+** | **-** | **-** | **-** | **-** |
|  | Chintamani, Karnataka, India | *Phaseolus vulgaris* | 1 | **-** | **-** | **-** | **+** | **-** | **-** | **-** | **-** |
|  | Chittoor, Andhra Pradesh, India | *Cajanus cajan* | 1 | **-** | **-** | **-** | **+** | **-** | **-** | **-** | **-** |
|  |  | *Macrotyloma uniflorum* | 1 | **-** | **-** | **-** | **+** | **-** | **-** | **-** | **-** |
|  |  | *Vigna mungo* | 2 | **+** | **-** | **-** | **-** | **-** | **-** | **-** | **-** |
|  | Chkkamagaluru, Karnataka, India | *Vigna radiata* | 1 | **-** | **+** | **-** | **-** | **-** | **-** | **-** | **-** |
|  | Coimbatore, Tamilnadu, India | *Lablab purpureus* | 4 | **-** | **-** | **+** | **-** | **-** | **-** | **-** | **-** |
|  |  | *Macrotyloma uniflorum* | 1 | **-** | **-** | **-** | **+** | **-** | **-** | **-** | **-** |
|  |  | *Vigna mungo* | 14 | **+** | **+** | **-** | **-** | **-** | **-** | **-** | **-** |
|  |  | *Vigna radiata* | 7 | **+** | **+** | **-** | **-** | **-** | **-** | **-** | **-** |
|  | Damoh, Madhya Pradesh, India | *Glycine max* | 1 | **+** | **-** | **-** | **-** | **-** | **-** | **-** | **-** |
|  | Dayalbagh, Uttar Pradesh, India | *Vigna mungo* | 1 | **+** | **-** | **-** | **-** | **-** | **-** | **-** | **-** |
|  | Dewas, Madhya Pradesh, India | *Vigna radiata* | 3 | **+** | **-** | **-** | **-** | **-** | **-** | **-** | **-** |
|  | Dharward, Karnataka, India | *Macrotyloma uniflorum* | 1 | **-** | **-** | **-** | **+** | **-** | **-** | **-** | **-** |
|  |  | *Vigna radiata* | 7 | **-** | **+** | **-** | **+** | **-** | **-** | **-** | **-** |
|  | Dholi, Bihar, India | *Vigna radiata* | 1 | **+** | **-** | **-** | **-** | **-** | **-** | **-** | **-** |
|  | Doddaballapur, Karnataka, India | *Phaseolus vulgaris* | 1 | **-** | **-** | **-** | **+** | **-** | **-** | **-** | **-** |
|  | Duddekunta, Andhra Pradesh, India | *Macrotyloma uniflorum* | 1 | **-** | **-** | **-** | **+** | **-** | **-** | **-** | **-** |
|  | East Godavari, Andhra Pradesh, India | *Vigna mungo* | 4 | **+** | **+** | **-** | **-** | **-** | **-** | **-** | **-** |
|  | Faizabad, Uttar Pradesh, India | *Vigna mungo* | 1 | **+** | **-** | **-** | **-** | **-** | **-** | **-** | **-** |
|  |  | *Vigna radiata* | 1 | **+** | **-** | **-** | **-** | **-** | **-** | **-** | **-** |
|  | Faridkot, Punjab, India | *Cajanus cajan* | 1 | **-** | **+** | **-** | **-** | **-** | **-** | **-** | **-** |
|  |  | *Vigna radiata* | 1 | **-** | **+** | **-** | **-** | **-** | **-** | **-** | **-** |
|  | Gadag, Karnataka, India | *Vigna radiata* | 1 | **-** | **+** | **-** | **-** | **-** | **-** | **-** | **-** |
|  | Gandhinagar, Gujarat, India | *Phaseolus vulgaris* | 1 | **-** | **-** | **-** | **-** | **+** | **-** | **-** | **-** |
|  | Guntur, Andhra Pradesh, India | *Abelmoschus moschatus* | 1 | **+** | **-** | **-** | **-** | **-** | **-** | **-** | **-** |
|  |  | *Cajanus cajan* | 1 | **-** | **-** | **-** | **+** | **-** | **-** | **-** | **-** |
|  |  | *Desmodium laxiflorum* | 1 | **+** | **-** | **-** | **-** | **-** | **-** | **-** | **-** |
|  |  | *Vigna mungo* | 7 | **+** | **-** | **-** | **-** | **-** | **-** | **-** | **-** |
|  |  | *Vigna radiata* | 1 | **+** | **-** | **-** | **-** | **-** | **-** | **-** | **-** |
|  | Gurdaspur, Punjab, India | *Vigna mungo* | 1 | **+** | **-** | **-** | **-** | **-** | **-** | **-** | **-** |
|  |  | *Vigna radiata* | 1 | **-** | **+** | **-** | **-** | **-** | **-** | **-** | **-** |
|  | Guwahati, Assam, India | *Vigna radiata* | 1 | **+** | **-** | **-** | **-** | **-** | **-** | **-** | **-** |
|  | Haldwani, Uttarakhand, India | *Vigna mungo* | 1 | **+** | **-** | **-** | **-** | **-** | **-** | **-** | **-** |
|  | Harda, Madhya Pradesh, India | *Vigna radiata* | 4 | **+** | **-** | **-** | **-** | **-** | **-** | **-** | **-** |
|  | Hisar, Haryana, India | *Duranta spp.* | 1 | **-** | **+** | **-** | **-** | **-** | **-** | **-** | **-** |
|  |  | *Vigna mungo* | 4 | **+** | **+** | **-** | **-** | **-** | **-** | **-** | **-** |
|  |  | *Vigna radiata* | 6 | **+** | **+** | **-** | **-** | **-** | **-** | **-** | **-** |
|  | Hoshangabad, Madhya Pradesh, India | *Vigna radiata* | 5 | **+** | **-** | **-** | **-** | **-** | **-** | **-** | **-** |
|  | Hoskote, Karnataka, India | *Phaseolus vulgaris* | 1 | **-** | **-** | **-** | **+** | **-** | **-** | **-** | **-** |
|  | Hyderabad, Telangana, India | *Pongamia pinnata* | 1 | **-** | **+** | **-** | **-** | **-** | **-** | **-** | **-** |
|  |  | *Vigna mungo* | 1 | **+** | **-** | **-** | **-** | **-** | **-** | **-** | **-** |
|  |  | *Vigna radiata* | 2 | **-** | **+** | **-** | **-** | **-** | **-** | **-** | **-** |
|  | Indore, Madhya Pradesh, India | *Bemisia tabaci* | 1 | **+** | **-** | **-** | **-** | **-** | **-** | **-** | **-** |
|  |  | *Glycine max* | 6 | **+** | **-** | **-** | **-** | **-** | **-** | **-** | **-** |
|  |  | *Lablab purpureus* | 1 | **-** | **-** | **+** | **-** | **-** | **-** | **-** | **-** |
|  |  | *Solanum melongena* | 1 | **+** | **-** | **-** | **-** | **-** | **-** | **-** | **-** |
|  |  | *Vigna radiata* | 3 | **+** | **-** | **-** | **-** | **-** | **-** | **-** | **-** |
|  | Jabalpur, Madhya Pradesh, India | *Cajanus cajan* | 1 | **+** | **-** | **-** | **-** | **-** | **-** | **-** | **-** |
|  |  | *Glycine max* | 6 | **+** | **+** | **-** | **-** | **-** | **-** | **-** | **-** |
|  |  | *Vigna mungo* | 1 | **+** | **-** | **-** | **-** | **-** | **-** | **-** | **-** |
|  | Jalandhar, Punjab, India | *Macrotyloma uniflorum* | 2 | **+** | **+** | **-** | **-** | **-** | **-** | **-** | **-** |
|  | Jalna, Maharashtra, India | *Glycine max* | 1 | **-** | **+** | **-** | **-** | **-** | **-** | **-** | **-** |
|  | Jhansi, Uttar Pradesh, India | *Vigna radiata* | 1 | **+** | **-** | **-** | **-** | **-** | **-** | **-** | **-** |
|  | Jodhpur, Rajasthan, India | *Vigna aconitifolia* | 1 | **+** | **-** | **-** | **-** | **-** | **-** | **-** | **-** |
|  |  | *Vigna radiata* | 2 | **+** | **-** | **-** | **-** | **-** | **-** | **-** | **-** |
|  | Jorhat, Assam, India | *Glycine max* | 2 | **+** | **+** | **-** | **-** | **-** | **-** | **-** | **-** |
|  | Kadapa, Andhra Pradesh, India | *Cajanus cajan* | 1 | **-** | **-** | **-** | **+** | **-** | **-** | **-** | **-** |
|  |  | *Vigna mungo* | 1 | **+** | **-** | **-** | **-** | **-** | **-** | **-** | **-** |
|  |  | *Vigna radiata* | 1 | **+** | **-** | **-** | **-** | **-** | **-** | **-** | **-** |
|  | Kalyani, West Bengal, India | *Clitoria ternatea* | 1 | **-** | **-** | **-** | **-** | **+** | **-** | **-** | **-** |
|  |  | *Glycine max* | 1 | **+** | **-** | **-** | **-** | **-** | **-** | **-** | **-** |
|  |  | *Lablab purpureus* | 1 | **-** | **-** | **+** | **-** | **-** | **-** | **-** | **-** |
|  | Kanpur, Uttar Pradesh, India | *Convolvulas arvensis* | 3 | **-** | **+** | **+** | **-** | **-** | **-** | **-** | **-** |
|  |  | *Lablab purpureus* | 3 | **+** | **-** | **+** | **-** | **-** | **-** | **-** | **-** |
|  |  | *Leonurus cardiaca* | 4 | **-** | **+** | **+** | **-** | **-** | **-** | **-** | **-** |
|  |  | *Medicago sativa* | 2 | **-** | **+** | **+** | **-** | **-** | **-** | **-** | **-** |
|  |  | *Phaseolus vulgaris* | 21 | **+** | **+** | **+** | **-** | **-** | **-** | **-** | **-** |
|  |  | *Rhynchosia minima* | 1 | **-** | **-** | **-** | **-** | **+** | **-** | **-** | **-** |
|  |  | *Vigna mungo* | 7 | **+** | **+** | **-** | **-** | **-** | **-** | **-** | **-** |
|  |  | *Vigna radiata* | 12 | **+** | **+** | **+** | **-** | **-** | **-** | **-** | **-** |
|  |  | *Vigna unguiculata* | 33 | **+** | **+** | **+** | **-** | **-** | **-** | **-** | **-** |
|  | Khatima, Uttarakhand, India | *Lablab purpureus* | 1 | **+** | **-** | **-** | **-** | **-** | **-** | **-** | **-** |
|  | Kolar, Karnataka, India | *Phaseolus vulgaris* | 1 | **-** | **-** | **-** | **+** | **-** | **-** | **-** | **-** |
|  | Kolkata, West Bengal, India | *Vigna mungo* | 1 | **+** | **-** | **-** | **-** | **-** | **-** | **-** | **-** |
|  | Krishna, Andhra Pradesh, India | *Vigna mungo* | 3 | **+** | **-** | **-** | **-** | **-** | **-** | **-** | **-** |
|  | Kurnool, Andhra Pradesh, India | *Cajanus cajan* | 1 | **-** | **-** | **-** | **+** | **-** | **-** | **-** | **-** |
|  |  | *Vigna mungo* | 4 | **+** | **-** | **-** | **-** | **-** | **-** | **-** | **-** |
|  | Ladhowal, Punjab, India | *Raya weed* | 1 | **-** | **+** | **-** | **-** | **-** | **-** | **-** | **-** |
|  |  | *Vigna radiata* | 2 | **-** | **+** | **-** | **-** | **-** | **-** | **-** | **-** |
|  | Lalitpur, Uttar Pradesh, India | *Glycine max* | 7 | **+** | **-** | **-** | **-** | **-** | **-** | **-** | **-** |
|  | Latur, Maharashtra, India | *Glycine max* | 1 | **+** | **-** | **-** | **-** | **-** | **-** | **-** | **-** |
|  |  | *Vigna unguiculata* | 1 | **-** | **+** | **-** | **-** | **-** | **-** | **-** | **-** |
|  | Lucknow, Uttar Pradesh, India | *Glycine max* | 1 | **+** | **-** | **-** | **-** | **-** | **-** | **-** | **-** |
|  |  | *Mucuna pruriens* | 1 | **-** | **-** | **-** | **-** | **-** | **-** | **-** | **+** |
|  | Ludhiana, Punjab, India | *Amaranthus viridis* | 2 | **-** | **+** | **-** | **-** | **-** | **-** | **-** | **-** |
|  |  | *Bemisia tabaci* | 2 | **+** | **-** | **-** | **-** | **-** | **-** | **-** | **-** |
|  |  | *Cannabis sativa* | 1 | **-** | **+** | **-** | **-** | **-** | **-** | **-** | **-** |
|  |  | *Commelina benghalensis* | 1 | **-** | **+** | **-** | **-** | **-** | **-** | **-** | **-** |
|  |  | *Cyperus rotundus* | 1 | **-** | **+** | **-** | **-** | **-** | **-** | **-** | **-** |
|  |  | *Digitaria sanguinalis* | 1 | **-** | **+** | **-** | **-** | **-** | **-** | **-** | **-** |
|  |  | *Glycine max* | 11 | **+** | **+** | **-** | **-** | **-** | **-** | **-** | **-** |
|  |  | *Launaea nudicaulis* | 1 | **-** | **+** | **-** | **-** | **-** | **-** | **-** | **-** |
|  |  | *Vigna mungo* | 6 | **+** | **+** | **-** | **-** | **-** | **-** | **-** | **-** |
|  |  | *Vigna radiata* | 12 | **+** | **+** | **-** | **-** | **-** | **-** | **-** | **-** |
|  |  | *Vigna unguiculata* | 1 | **+** | **-** | **-** | **-** | **-** | **-** | **-** | **-** |
|  | Madurai, Tamilnadu, India | *Glycine max* | 2 | **-** | **+** | **-** | **-** | **-** | **-** | **-** | **-** |
|  | Malur, Karnataka, India | *Phaseolus vulgaris* | 1 | **-** | **-** | **-** | **+** | **-** | **-** | **-** | **-** |
|  | Meerut, Uttar Pradesh, India | *Vigna radiata* | 1 | **+** | **-** | **-** | **-** | **-** | **-** | **-** | **-** |
|  |  | *Vigna unguiculata* | 2 | **+** | **-** | **-** | **-** | **-** | **-** | **-** | **-** |
|  | Mettur, Tamilnadu, India | *Capsicum annuum* | 1 | **-** | **-** | **+** | **-** | **-** | **-** | **-** | **-** |
|  | Moga, Punjab, India | *Vigna radiata* | 1 | **-** | **+** | **-** | **-** | **-** | **-** | **-** | **-** |
|  | Muktsar, Punjab, India | *Vigna radiata* | 1 | **-** | **+** | **-** | **-** | **-** | **-** | **-** | **-** |
|  | Mulbagal, Karnataka, India | *Phaseolus vulgaris* | 1 | **-** | **-** | **-** | **+** | **-** | **-** | **-** | **-** |
|  | Mysore, Karnataka, India | *Lablab purpureus* | 1 | **-** | **-** | **+** | **-** | **-** | **-** | **-** | **-** |
|  | Nagpur, Maharashtra, India | *Glycine max* | 1 | **-** | **+** | **-** | **-** | **-** | **-** | **-** | **-** |
|  | Namakkal, Tamilnadu, India | *Vigna aconitifolia* | 2 | **-** | **+** | **-** | **-** | **-** | **-** | **-** | **-** |
|  |  | *Vigna radiata* | 4 | **+** | **+** | **-** | **-** | **-** | **-** | **-** | **-** |
|  | Narsinghpur, Madhya Pradesh, India | *Solanum melongena* | 1 | **+** | **-** | **-** | **-** | **-** | **-** | **-** | **-** |
|  | Navsari, Gujarat, India | *Cajanus cajan* | 1 | **-** | **-** | **-** | **-** | **+** | **-** | **-** | **-** |
|  |  | *Glycine max* | 1 | **-** | **+** | **-** | **-** | **-** | **-** | **-** | **-** |
|  |  | *Vigna radiata* | 1 | **-** | **+** | **-** | **-** | **-** | **-** | **-** | **-** |
|  | Nelamangala, Karnataka, India | *Phaseolus vulgaris* | 1 | **-** | **-** | **-** | **+** | **-** | **-** | **-** | **-** |
|  | Nellore, Andhra Pradesh, India | *Vigna mungo* | 2 | **+** | **-** | **-** | **-** | **-** | **-** | **-** | **-** |
|  | New Delhi, India | *Bemisia tabaci* | 1 | **+** | **-** | **-** | **-** | **-** | **-** | **-** | **-** |
|  |  | *Cajanus cajan* | 1 | **+** | **-** | **-** | **-** | **-** | **-** | **-** | **-** |
|  |  | *Cassia fistula* | 1 | **+** | **-** | **-** | **-** | **-** | **-** | **-** | **-** |
|  |  | *Glycine max* | 8 | **+** | **-** | **-** | **-** | **-** | **-** | **-** | **-** |
|  |  | *Lablab purpureus* | 4 | **-** | **-** | **+** | **-** | **-** | **-** | **-** | **-** |
|  |  | *Vigna mungo* | 1 | **-** | **+** | **-** | **-** | **-** | **-** | **-** | **-** |
|  |  | *Vigna radiata* | 16 | **+** | **+** | **-** | **-** | **-** | **-** | **-** | **-** |
|  |  | *Vigna unguiculata* | 3 | **+** | **-** | **-** | **-** | **-** | **-** | **-** | **-** |
|  | Obaidullaganj, Madhya Pradesh, India | *Vigna radiata* | 2 | **+** | **-** | **-** | **-** | **-** | **-** | **-** | **-** |
|  | Ongole, Andhra Pradesh, India | *Vigna mungo* | 1 | **+** | **-** | **-** | **-** | **-** | **-** | **-** | **-** |
|  | Palampur, Himachal Pradesh, India | *Phaseolus vulgaris* | 1 | **+** | **-** | **-** | **-** | **-** | **-** | **-** | **-** |
|  | Pantnagar, Uttarakhand, India | *Alternanthera sessilis* | 1 | **+** | **-** | **-** | **-** | **-** | **-** | **-** | **-** |
|  |  | *Glycine max* | 3 | **+** | **-** | **-** | **-** | **-** | **-** | **-** | **-** |
|  |  | *Lablab purpureus* | 1 | **+** | **-** | **-** | **-** | **-** | **-** | **-** | **-** |
|  |  | *Vigna radiata* | 1 | **+** | **-** | **-** | **-** | **-** | **-** | **-** | **-** |
|  | Patna, Bihar, India | *Vigna unguiculata* | 1 | **+** | **-** | **-** | **-** | **-** | **-** | **-** | **-** |
|  | Phanda, Uttar Pradesh, India | *Vigna mungo* | 1 | **+** | **-** | **-** | **-** | **-** | **-** | **-** | **-** |
|  |  | *Vigna radiata* | 2 | **+** | **-** | **-** | **-** | **-** | **-** | **-** | **-** |
|  | Prakasam, Andhra Pradesh, India | *Abelmoschus moschatus* | 1 | **+** | **-** | **-** | **-** | **-** | **-** | **-** | **-** |
|  |  | *Cajanus cajan* | 1 | **-** | **-** | **-** | **+** | **-** | **-** | **-** | **-** |
|  |  | *Desmodium laxiflorum* | 1 | **+** | **-** | **-** | **-** | **-** | **-** | **-** | **-** |
|  |  | *Vigna mungo* | 3 | **+** | **-** | **-** | **-** | **-** | **-** | **-** | **-** |
|  | Raichur, Karnataka, India | *Vigna mungo* | 6 | **+** | **+** | **-** | **+** | **-** | **-** | **-** | **-** |
|  |  | *Vigna stipulacea* | 3 | **+** | **+** | **-** | **+** | **-** | **-** | **-** | **-** |
|  |  | *Vigna unguiculata* | 1 | **+** | **-** | **-** | **-** | **-** | **-** | **-** | **-** |
|  | Raipur, Chhattisgarh, India | *Cajanus scarabaeoides* | 1 | **-** | **-** | **-** | **-** | **-** | **-** | **+** | **-** |
|  |  | *Glycine max* | 3 | **+** | **-** | **-** | **-** | **-** | **-** | **-** | **-** |
|  |  | *Vigna radiata* | 1 | **+** | **-** | **-** | **-** | **-** | **-** | **-** | **-** |
|  |  | *Vigna unguiculata* | 3 | **+** | **-** | **-** | **-** | **-** | **-** | **-** | **-** |
|  | Ranchi, Jharkhand, India | *Vigna unguiculata* | 1 | **+** | **-** | **-** | **-** | **-** | **-** | **-** | **-** |
|  | Ranibennur, Karnataka, India | *Cyamopsis tetragonoloba* | 1 | **-** | **+** | **-** | **-** | **-** | **-** | **-** | **-** |
|  | Rekulakunta, Andhra Pradesh, India | *Macrotyloma uniflorum* | 1 | **-** | **-** | **-** | **+** | **-** | **-** | **-** | **-** |
|  | Sagar, Madhya Pradesh, India | *Glycine max* | 1 | **+** | **-** | **-** | **-** | **-** | **-** | **-** | **-** |
|  |  | *Solanum melongena* | 1 | **+** | **-** | **-** | **-** | **-** | **-** | **-** | **-** |
|  | Salem, Tamilnadu, India | *Lablab purpureus* | 2 | **-** | **-** | **+** | **-** | **-** | **-** | **-** | **-** |
|  | Samastipur, Bihar, India | *Lablab purpureus* | 1 | **-** | **-** | **+** | **-** | **-** | **-** | **-** | **-** |
|  | Sangrur, Punjab, India | *Vigna radiata* | 1 | **-** | **+** | **-** | **-** | **-** | **-** | **-** | **-** |
|  | Satna, Madhya Pradesh, India | *Clitoria ternatea* | 1 | **+** | **-** | **-** | **-** | **-** | **-** | **-** | **-** |
|  |  | *Cucurbita maxima* | 1 | **+** | **-** | **-** | **-** | **-** | **-** | **-** | **-** |
|  |  | *Glycine max* | 10 | **+** | **-** | **-** | **-** | **-** | **-** | **-** | **-** |
|  |  | *Phaseolus vulgaris* | 1 | **+** | **-** | **-** | **-** | **-** | **-** | **-** | **-** |
|  |  | *Solanum lycopersicum* | 1 | **+** | **-** | **-** | **-** | **-** | **-** | **-** | **-** |
|  | Sehore, Madhya Pradesh, India | *Glycine max* | 16 | **+** | **-** | **-** | **-** | **-** | **-** | **-** | **-** |
|  |  | *Vigna radiata* | 4 | **+** | **-** | **-** | **-** | **-** | **-** | **-** | **-** |
|  | Shillong, Meghalaya, India | *Vigna radiata* | 1 | **+** | **-** | **-** | **-** | **-** | **-** | **-** | **-** |
|  | Shivamogga, Karnataka, India | *Vigna radiata* | 2 | **-** | **+** | **-** | **-** | **-** | **-** | **-** | **-** |
|  | Sidlagatta, Karnataka, India | *Phaseolus vulgaris* | 1 | **-** | **-** | **-** | **+** | **-** | **-** | **-** | **-** |
|  | Solapur, Maharashtra, India | *Glycine max* | 1 | **+** | **-** | **-** | **-** | **-** | **-** | **-** | **-** |
|  | Srinivaspura, Karnataka, India | *Phaseolus vulgaris* | 1 | **-** | **-** | **-** | **+** | **-** | **-** | **-** | **-** |
|  | Sultanpur, Uttar Pradesh, India | *Cajanus cajan* | 1 | **+** | **-** | **-** | **-** | **-** | **-** | **-** | **-** |
|  | Surat, Gujarat, India | *Lablab purpureus* | 1 | **-** | **-** | **+** | **-** | **-** | **-** | **-** | **-** |
|  |  | *Vigna radiata* | 1 | **+** | **-** | **-** | **-** | **-** | **-** | **-** | **-** |
|  | Thiruvananthapuram, Kerala, India | *Rhynchosia minima* | 2 | **-** | **-** | **-** | **-** | **-** | **+** | **-** | **-** |
|  | Tirunelveli, Tamilnadu, India | *Vigna mungo* | 1 | **-** | **+** | **-** | **-** | **-** | **-** | **-** | **-** |
|  | Tirupati, Andhra Pradesh, India | *Annona squamosa* | 1 | **+** | **-** | **-** | **-** | **-** | **-** | **-** | **-** |
|  |  | *Cajanus cajan* | 1 | **-** | **-** | **-** | **+** | **-** | **-** | **-** | **-** |
|  |  | *Calotropis gigantea* | 1 | **+** | **-** | **-** | **-** | **-** | **-** | **-** | **-** |
|  |  | *Jatropha curcas* | 1 | **+** | **-** | **-** | **-** | **-** | **-** | **-** | **-** |
|  |  | *Lablab purpureus* | 1 | **-** | **-** | **+** | **-** | **-** | **-** | **-** | **-** |
|  |  | *Macrotyloma uniflorum* | 5 | **-** | **-** | **-** | **+** | **-** | **-** | **-** | **-** |
|  |  | *Pedalium murex* | 1 | **+** | **-** | **-** | **-** | **-** | **-** | **-** | **-** |
|  |  | *Vigna mungo* | 12 | **+** | **+** | **-** | **-** | **-** | **-** | **-** | **-** |
|  |  | *Vigna radiata* | 1 | **-** | **+** | **-** | **-** | **-** | **-** | **-** | **-** |
|  | Triuvallur, Tamilnadu, India | *Vigna mungo* | 1 | **+** | **-** | **-** | **-** | **-** | **-** | **-** | **-** |
|  | Tumakuru, Karnataka, India | *Achyranthes aspera* | 1 | **-** | **+** | **-** | **-** | **-** | **-** | **-** | **-** |
|  |  | *Amaranthus viridis* | 1 | **-** | **-** | **-** | **+** | **-** | **-** | **-** | **-** |
|  |  | *Croton bonplandianus* | 1 | **-** | **-** | **-** | **+** | **-** | **-** | **-** | **-** |
|  |  | *Sida rhombifolia* | 1 | **-** | **-** | **-** | **+** | **-** | **-** | **-** | **-** |
|  |  | *Tephrosia purpurea* | 1 | **-** | **-** | **-** | **+** | **-** | **-** | **-** | **-** |
|  | Utukur, Andhra Pradesh, India | *Macrotyloma uniflorum* | 1 | **-** | **-** | **-** | **+** | **-** | **-** | **-** | **-** |
|  | Vamban, Tamilnadu, India | *Vigna mungo* | 10 | **+** | **+** | **-** | **-** | **-** | **-** | **-** | **-** |
|  |  | *Vigna radiata* | 5 | **+** | **+** | **-** | **-** | **-** | **-** | **-** | **-** |
|  | Varanasi, Uttar Pradesh, India | *Lablab purpureus* | 1 | **+** | **-** | **-** | **-** | **-** | **-** | **-** | **-** |
|  |  | *Phaseolus vulgaris* | 3 | **+** | **-** | **-** | **-** | **-** | **-** | **-** | **-** |
|  |  | *Vigna mungo* | 1 | **+** | **-** | **-** | **-** | **-** | **-** | **-** | **-** |
|  |  | *Vigna radiata* | 1 | **+** | **-** | **-** | **-** | **-** | **-** | **-** | **-** |
|  |  | *Vigna unguiculata* | 1 | **+** | **-** | **-** | **-** | **-** | **-** | **-** | **-** |
|  | Vizianagaram, Andhra Pradesh, India | *Vigna mungo* | 2 | **+** | **-** | **-** | **-** | **-** | **-** | **-** | **-** |
|  | West Godavari, Andhra Pradesh, India | *Vigna mungo* | 2 | **+** | **-** | **-** | **-** | **-** | **-** | **-** | **-** |

***Legumovirus**: MYMIV*=Mungbean yellow mosaic India virus,* MYMV*= Mungbean yellow mosaic virus,* DoYMV*= Dolichos yellow mosaic virus,* HgYMV*= Horsegram yellow mosaic virus,* RhYMV*= Rhynchosia yellow mosaic virus,* RhYMIV*= Rhynchosia yellow mosaic India virus,* CsYMV*= Cajanus scarabaeoides yellow mosaic virus,* VbSMV*= Velvet bean severe mosaic virus.*
